# In-depth comparison of commercial *Trichoderma-*based products: integrative approaches to quantitative analysis, taxonomy and efficacy

**DOI:** 10.3389/fmicb.2025.1646394

**Published:** 2025-09-18

**Authors:** Tomasz Kulik, Paulina Staniszewska, Patryk Wiśniewski, Zofia Treder, Maciej Przybylski, Ewa Wrońska, Mateusz Maździarz, Katarzyna Krawczyk, Katarzyna Bilska, Łukasz Paukszto, Jacek Olszewski

**Affiliations:** ^1^Department of Botany and Evolutionary Ecology, University of Warmia and Mazury in Olsztyn, Olsztyn, Poland; ^2^Experimental Education Unit, University of Warmia and Mazury in Olsztyn, Olsztyn, Poland

**Keywords:** *Trichoderma*, biopesticides, biofertilizers, biocontrol, biofungicides

## Abstract

Products derived from *Trichoderma* fungi, primarily marketed as biofungicides and biofertilizers, are widely utilized to promote sustainable and regenerative farming practices. In this study, we characterized *Trichoderma*-based products currently available in both international and local (the Polish) markets. We estimated the number of viable cells in these products, which is a key factor boosting their efficacy. We found substantial differences in the number of colony-forming units (CFUs) among various products, with all products exhibiting fewer CFUs than claimed. The degree of this inconsistency was notably heightened in the case of biofertilizers. We also determined the species identity of isolates recovered from these products using MIST approach. For most products, results of the multilocus species identification revealed inconsistency with taxonomic classification available on product labels or failed to confirm their taxonomic status. We also assessed variation in the invasion capacity of *Trichoderma* isolates against economically important plant pathogens *B. cinerea, F. graminearum* and *S. sclerotiorum* using *in vitro* approaches. To address the identified challenges associated with the suboptimal quality of biofertilizers, various targeted solutions are discussed and proposed.

## 1 Introduction

Growing interest in environmentally friendly practices in crop production has contributed to the increased use of beneficial microorganisms, including fungi. As plant production undergoes ongoing transformation, it is expected that the role of biopesticides in crop management will become increasingly important (Galli et al., 2024). The FAO and WHO actively promote responsible pesticide management, increasingly focusing on biopesticides (Schaffner et al., 2024). While synthetic pesticides dominate the market, projected to reach $139.42 billion by 2027, representing a compound annual growth rate (CAGR) of 9.1%, the biopesticides market demonstrates stronger growth potential. It is expected to reach $13.9 billion by 2028, with a high CAGR of 15.9%. Given that over 55% of global biopesticides are microbial, their significant value is evident (Wend et al., 2024).

It is worth noting that the regulatory landscape for biological products, influenced by European Union harmonization efforts, varies by country and features distinct guidelines that primarily focus on safety, efficacy, and ecological factors (Chandler et al., 2011). Fungal-derived products play a crucial role in sustainable and regenerative agriculture and are mostly available in the market as biofungicides and biofertilizers (syn. biostimulants) (Awad-Allah et al., 2022). Biofungicides are tailored to control pathogens and work best when applied preventively before disease development (Yao et al., 2023). Biofertilizers are generally designed to provide numerous benefits to the soil, directly or indirectly accelerating plant growth and improving the stress tolerance of crops (Mahanty et al., 2017). While *Trichoderma* strains are approved as active substances for pesticides under EU Regulation (EC) no 1107/2009, their status as biofertilizers is more complex. The EU’s harmonized list for microbial biostimulants (Component Material Category 7) is currently limited to genera such as *Azotobacter* and *Rhizobium*, excluding *Trichoderma*. This means *Trichoderma* biostimulants rely on national regulations (Chandler et al., 2011), such as those in Poland, where authorization from the Minister of Agriculture requires extensive efficacy data. Organizations like the European Biostimulants Industry Council (EBIC) are lobbying for the expansion of this EU list (European Biostimulant Industry Council, 2025; European Union, 2025)

The genus *Trichoderma* displays tremendous diversity in morphological, physiological, and biochemical properties. The impressive taxonomic diversity of this genus is also apparent among various commercial strains. Beneficial strains, including *Trichoderma afroharzianum*, *T. asperellum*, *T. atroviride*, *T. gamsii*, *T. longibrachiatum*, *T. reesei*, and *T. viride*, have been marketed on both national and international scales (Meher et al., 2020; Tyśkiewicz et al., 2022; Guzmán-Guzmán et al., 2023). The distinct strain-specific properties, such as growth rate, sporulation capacity, environmental adaptability, biochemical traits, and mycoparasitic activity, may reflect variations in the performance of fungal-derived products. Besides the unique properties of the strains, the formulation of biological products including the number of viable cells, type of carrier materials, and their stability, plays a significant role in influencing performance (Błaszczyk et al., 2014).

In this study, we sought to characterize *Trichoderma*-based products currently available in both international and local (the Polish) markets. We estimated the number of viable fungal cells in these products, as a vital feature that directly affects their efficacy (Mulatu et al., 2021). We also validated the species identity of strains recovered from these products using MIST approach. Finally, to detect variation in their biocontrol performance, we measured the ability of *Trichoderma* isolates to parasite inoculum of economically important *Botrytis cinerea, Fusarium graminearum* and *Sclerotinia sclerotiorum.*

## 2 Materials and methods

### 2.1 *Trichoderma*-based products

*Trichoderma-*based products intended for use in plant production were purchased online from commercial suppliers in Poland. After arrival at the laboratory, they were stored in the refrigerator at 4 °C. More detailed characteristics of these products are shown in Table 1.

**Table 1:** *Trichoderma*-based products used in this study.

Commercial name	Producer	Claimed taxonomic status (species, strain)	Formulation and recommended doses (kg/ha)	Authorized^c^
Biocontrol T-34^a^	Biocontrol Technologies, S. L., Avgda. Madrid, 215–217, entresol A, 0814 Barcelona, Spain	*T. asperellum*, T34	powder, 0.25–0.5kg/ha	AT, BE, CY, CZ, DE, DK, EL, ES, FR, HU, IE, IT, LV, NL, PL, PT, RO, SE, SI, SK
Vintec^a^	Certis Belchim BV Technologielaan 7 B-1840 Londerzeel, Belgium	*T. atroviride,* SC1	granular, 0.2kg/ha	AT, BE, CY, CZ, DE, EL, ES, FR, HR, HU, IT, LU, NL, PL, PT, SI, SK
Trianum-G^a^	Koppert BV, Veilingweg 14, 2651 BE Berkel en Rodenrijs, The Netherlands	*T. harzianum*, T-22	granular, 10–50 kg/ha	BE, BG, CZ, ES, FI, HR, HU, IE, IT, LV, NL, PT, SE
Remedier^a^	ISAGRO S.p. A. Centro Uffizi San Siro – Edificio D- ala 3-Via Caldera 21–20153 Milan, Italy	*T. asperellum*, ICC 012 and *T. gamsii*, ICC 080	powder, 2.5kg/ha	CY, DE, EL, ES, MT, NL, PL, PT
Trichofit^b^	Biofeld Sp. z o. o. ul. Roźwienicka 43, 37–500 Widna Góra, Poland	*Trichoderma*	powder, 0.3–0.5kg/ha	PL
Trichoderma^b^	Gospodarstwo Badawczo-Rozwojowe Robert Cysewski. ul. Błażeja 78/4 61–608 Poznań, Poland	*T. atrobrunneum, T. koningii, T. atroviride*	liquid, 0.4–1.2l/ha	PL
Trichogel^b^	BTU -CENTER Europe GmbH Willesch 3, 49779 Oberlangen, Germany	*T. harzianum, T. viride, T. reesei, T. koningii*	gel, 1-3l/ha	PL
Mocne Korzenie (MK)^b^	BHUMI Sp. z o.o. ul. Ilji Miecznikowa 1/305D, 02–096 Warsaw, Poland	*T. harzianum, T. viride*	dextrose powder, 0.25kg/ha	PL

a Biofungicide.

b Biofertilizer.

c Strain authorization according to EU Pesticides database (https://food.ec.europa.eu/plants/pesticides/eu-pesticides-database_en).

AT – Austria, BE – Belgium, BG – Bulgaria, CY – Cyprus, CZ – Czech Republic, DE – Germany, DK – Denmark, EL – Greece, ES – Spain, FI – Finland, FR – France, HU – Hungary, IE – Ireland, IT – Italy, LU – Luxembourg, LV – Latvia, MT – Malta, NL – Netherlands, PL – Poland, PT – Portugal, RO – Romania, SE – Sweden, SI – Slovenia, SK – Slovakia.

### 2.2 Colony-forming unit assays

The samples of *Trichoderma-*based products (0.5g from powder products and 0.5ml from liquid/gel formulations) were diluted in distilled water (up to 10^−9^ of each original sample) and 1mL of each dilution was plated on Potato Dextrose Agar with streptomycin (30 mg/L) and penicillin (100 mg/L). For each product, three independent biological replicates were carried out. PDA plates were incubated at 25 °C for 3 days at which time colony forming units (CFU) were calculated. For further analysis, isolates were plated on single PDA plates and incubated at 25 °C for 7 days. For isolates obtained from the same biological product, isolates with distinct morphologies were selected for whole-genome sequencing (WGS).

### 2.3 DNA extraction and whole-genome sequencing

For DNA extraction from isolates grown on agar medium, 0.1 g of mycelium was scraped with a sterile tip from fungi grown on PDA for 7 days at 25 °C. Total DNA ranging from 3 to13 μg from each isolate was quantified on a Qubit fluorometer using a Qubit dsDNA BR assay kit (Applied Biosystems, Foster City, CA, United States). Whole-genome sequencing was performed as previously described by Kulik et al. (2022). Briefly, genomes of all strains were sequenced by Macrogen (Seoul, South Korea) on an Illumina HiSeq X Ten using a paired-end read length of 2 × 150 bp with an insert size of 350 bp. Libraries were prepared using TruSeq DNA PCR-free library preparation kit (Illumina, San Diego, CA, United States). All genomic data generated herein were deposited in the NCBI Sequence Read Archive under GenBank accession no. PRJNA1017172. The genomes were further assembled via SPAdes (v.3.13.2) (Nurk et al., 2013) with k-mer values of 21, 33, 55, 77, 99, and 127 and using the “careful” option to reduce mismatches.

### 2.4 Taxonomic assignments of the strains using MIST approach

For taxonomic assignments, complete sequences of barcodes: TEF1 (translation elongation factor 1 alpha), RPB2 (RNA polymerase II genes) and ITS (Internal Transcribed Spacer) were retrieved from the genome assembly of studied strains based on a homology search for the corresponding sequences from the strain (*T. reesei,* Li et al., 2017) with Geneious Prime software (Biomatters Ltd., New Zealand). *Trichoderma* species were identified using the MIST database according the protocol from Dou et al. (2020) by comparing TEF1, RPB2 and ITS sequences (Supplementary Table 1). A match required ≥95% identity for each gene. If the ITS sequence matched multiple species at 95%, the analysis was repeated with a ≥ 99% identity threshold.

### 2.5 Plate confrontation assays

The biocontrol activity of *Trichoderma* isolates against *B. cinerea, F. graminearum and S. sclerotiorum* was assessed by confrontation coculture. The assays included four strains of *Trichoderma* (T34, SC1, T-22 and ICC012) recovered from biofungicides. In addition, based on the product data sheets of two biofertilizers: Trichofit and Trichoderma, which indicated the antifungal and mycoparasitic activity of their strains, we decided to include three strains (Tf1, Cys1 and Cys2) obtained from these two biofertilizers.

Mycelia plugs from *Trichoderma* plates and individual pathogens, each 10 mm in diameter and cut from the margin of the fungal colonies grown on PDA medium were placed on a 90 mm PDA dish (a distance of 45 mm from each other). In control group, the plug containing pathogen hyphae was faced with an agar plug. Three replicates were set for the controls and the *Trichoderma-*pathogen combinations. All the plates were cultured at 25 °C for three weeks. The radial growth of pathogens was measured in the presence (treatment) or absence (controls) of *Trichoderma*. Growth inhibition percentage was calculated as follows: RGI% = (RC − RT)/RC × 100, where RGI is the percentage of radial growth inhibition, RC is the radial growth (mm) of the pathogen (controls) and RT = radial growth (mm) of pathogen grown in the presence of *Trichoderma* isolates.

Isolates exhibiting full overgrowth and sporulation over pathogen hyphae, were assigned as 100% effective against pathogens.

### 2.6 Statistical analyses

The significance of the growth reduction was checked with a Kruskal-Wallis test, and a post-hoc Dunn’s test was performed. Differences in isolates with an adjusted *p*-value less than 0.05 were considered statistically significant.

## 3 Results

**Figure 1:**
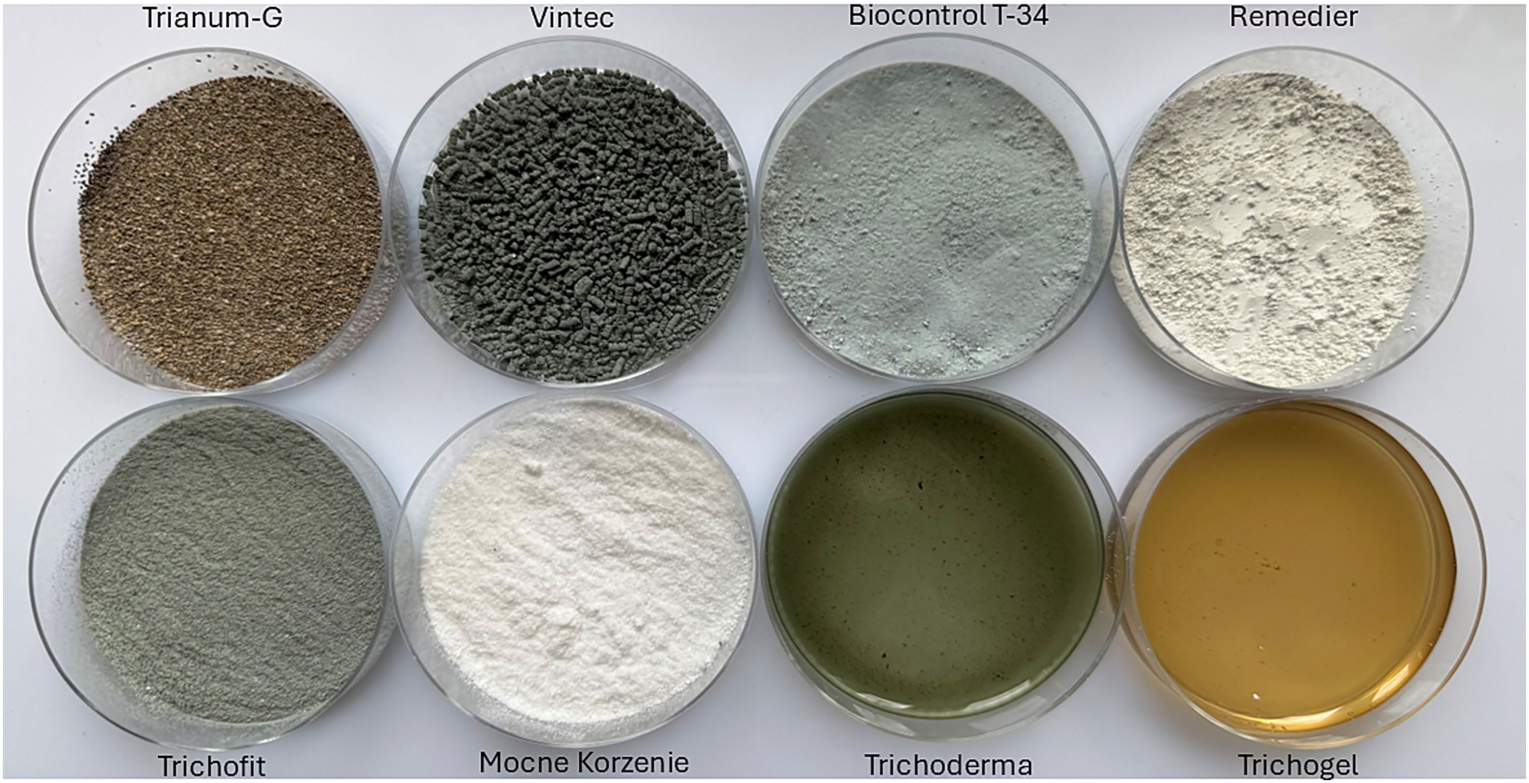
*Trichoderma*-based products formulated in both solid and liquid forms evaluated in this study.

In this study, commercial *Trichoderma*-based products formulated in both solid and liquid forms were evaluated. Powder-based products showed differences in granularity, ranging from fine powders to water-dispersible granules. The products varied in color, ranging from mustard and olive green to dark green. The exception was the product Mocne Korzenie, which was white in color (Figure 1).

### 3.1 The quantification of CFUs and colony characteristics of isolates

Among studied biofungicides, the estimated number of viable fungal cells in *Trichoderma* products ranged from 1.5 × 10^9^ to 2.3 × 10^7^ CFUs (Table 2). Biofertilizers showed higher variation in CFUs, ranging from 6.4 × 10^8^ to only 1 × 10^2^ CFUs. In the case of Mocne Korzenie, CFU assays did not yield fungal growth on the PDA medium. All tested products exhibited lower CFU counts than those claimed on labels.

**Table 2:** Claimed and quantified CFUs in *Trichoderma* products.

Commercial name	Isolate	Estimated CFU/g (SD)	Claimed CFU/g
Biocontrol T-34^a^	T-34	3 × 10^8^ (1.6)	1 × 10^9^
Vintec^a^	SC1	1.5 × 10^9^ (5.4)	9 × 10^9^
Trianum-G^a^	T-22	3.7 × 10^7^ (2.5)	1.5 × 10^8^
Remedier^a^	ICC 012	2.3 × 10^7^ (1.2)	undetermined
Trichofit^b^	Tf1	6.4 × 10^8^ (2.7)	2 × 10^9^
Trichoderma^b^	Cys1 Cys2	2.7 × 10^7^ (0.9) 6 × 10^7^ (0.8)	2 × 10^10^
Trichogel^b^	Tg1 Tg2	1 × 10^2^ (0) 1 × 10^2^ (0)	undetermined
Mocne korzenie (MK)^b^	No fungal growth	Undetermined	10^8^ *T. harzianum*, 10^8^ *T. viride*

a Biofungicide.

b Biofertilizer.

Preliminary morphological examination of colony morphology of isolates revealed that all recovered isolates exhibited the morphological characteristics of *Trichoderma*, including white filaments and green to olive-green spores (data not shown). Two biofertlizers, Trichoderma and Trichogel, yielded isolates with two distinct morphologies, which contrasts with the claimed three to four-strain composition of these products. In addition, our results did not support the multi-strain composition of Remedier, as this product yielded isolates exhibiting the same morphological characteristics.

Isolates recovered from biofungicides were named according to the names of the strains indicated on the product labels (Table 1). Isolates recovered from biofertilizers were assigned as follows: Tf1 (from Trichofit), Cys1 and Cys2 (from Trichoderma biofertilizer), Tg1 and Tg2 (from Trichogel) and MK1 (from Mocne Korzenie).

### 3.2 Whole-genome sequencing and extraction of barcodes for determination of species identity of the recovered isolates

Whole genome sequencing yielded a total of 10 SRA (Sequence Read Archive) submissions (SAMN47809852–SAMN47809861), containing over 13.3 Gb of raw sequence data. For taxonomic assignments, nucleotide sequences of TEF1, RPB2 and ITS were successfully retrieved from all genome assembly (Supplementary Table 1).

### 3.3 Taxonomic status of isolates recovered from *Trichoderma*–based products

In an attempt to identify the taxonomic status of the recovered isolates, we used the online multilocus identification system (MIST) for automated identification of *Trichoderma* isolates (Dou et al., 2020). MIST is based on the reference databases of validated sequences of three commonly used DNA barcodes: TEF1, RPB2 and ITS. Among ten isolates only four were determined to single species. Isolate T-34, Cys1 and Tg2 were identified as *T. asperellum.* Isolate Cys2 was determined as *T. atrobrunneum*. Results of the MIST analysis assigned the remaining isolates multiple species names: SC1 as *T. atroviride* and *T. viride*; T-22 as *T. afroharzianum*, *T. atrobrunneum*, *T. breve*, and *T. simmonsii*; ICC 012 as *T. asperelloides*, *T. asperellum*, and *T. pubescens*; Tf1 as *T. aethiopicum*, *T. longibrachiatum*, and *T. orientale*; Tg1 as *T. afarasin*, *T. atrobrunneum*, *T. breve*, *T. endophyticum*, *T. harzianum*, *T. rifaii*, *T. simmonsii*, *T. zeloharzianum*; and MK1 as *T. aethiopicum*, *T. longibrachiatum*, and *T. orientale.*

The obtained results of the analysis were inconsistent with taxonomic classification available on product labels. The single exception was T-34 isolate, which was consistently identified according to the product description as *T. asperellum*. Notably, the results of MIST analysis of T-22 isolate recovered from Trianum-G did not support current reclassification of this strain as *T. afroharzianum* (Chaverri et al., 2015).

### 3.4 Plate confrontation assays

Plate confrontation assays allowed for the detection of the invading capacity of the tested *Trichoderma* isolates (Supplementary Figure 1). A high success rate of invasive activity, resulting from the full overgrowth of pathogen hyphae, was observed in all but one dual-cultures with isolates recovered from biofungicides. The exceptional isolate, T-22, did not overgrow *F. graminearum* hyphae but inhibited its growth by more than half. Among the isolates recovered from biofertilizers, one (Cys1) completely overgrew the biomass of all tested pathogens. In most dual cultures with the remaining isolates, Tfl and Cys2, a lack of or weak mycoparasitic activity was observed. In these samples, different degrees of growth reduction of pathogen hyphae were found (Table 3; Supplementary Table 2).

**Table 3:** Variation in the activity of *Trichoderma* isolates against pathogens.

Isolation source	Isolate	Growth reduction (%)		
		*B. cinerea*	*F. graminearum*	*S. sclerotiorum*
Biocontrol T-34^1^	T-34	100	100	100
Vintec^a^	SC1	100	100	100
Trianum-G^a^	T-22	100	53.7 (SD 3.23)	100
Remedier^a^	ICC 012	100	100	100
Trichofit^b^	Tf1	63.9 (SD 3.0)	57.8 (SD 2.0)	53.7 (SD 3.2)
Trichoderma^b^	Cys1	100	100	100
	Cys2	41.5 (SD 9.7)	20.7 (SD 3.2)	41.5 (SD 9.7)

a Biofungicide.

b Biofertilizer.

(100) indicates an invasive interaction leading to the complete overgrowth of pathogen hyphae by Trichoderma. Values below (100) indicate the percentage of growth reduction of pathogen hyphae. In these samples, a lack of or weak mycoparasitic activity was observed. Data were expressed as mean ± standard deviation (SD).

## 4 Discussion

Fungi of the genus *Trichoderma* have gained significant attention in agriculture due to their multifaceted roles as biocontrol agents, enhancers of soil health, and promoters of plant growth. A variety of products harnessing these beneficial fungi have been commercially developed, leading to a prominent position in the global market (Tyśkiewicz et al., 2022). One key aspect of product labeling is the viability of spores, commonly represented as CFUs (Mulatu et al., 2021). In our study, we found that all analyzed products contained a lower number of CFUs than advertised, with biofungicides displaying discrepancies ranging from 3.3 to 5 times fewer CFUs than claimed. Such discrepancies may be attributed to issues surrounding the limited shelf life and inherent instability commonly associated with biological products (Martinez et al., 2023). Notably, this inconsistency was exacerbated in biofertilizers, where two out of four products demonstrated a dramatic reduction in CFUs.

Beyond the intrinsic properties of the *Trichoderma* strains themselves, the formulation of biological products encompassing the type of carrier materials and their inherent stability plays a significant role in influencing overall product performance. The observed widespread discrepancies in CFUs across all tested products, notably the dramatic reduction found in biofertilizers, strongly indicate potential issues related to formulation stability and the suitability of the chosen vehicles. These factors are critical for ensuring both the long-term shelf life of the product and its consistent application efficiency in agricultural settings, as inadequate production and storage facilities can present significant challenges to maintaining high-quality inoculants.

Another critical component of labeling is the use of binomial nomenclature, which includes the scientific names (genus and species) of active ingredients. Scientific nomenclature serves to eliminate ambiguity, facilitating effective communication among stakeholders. However, our study revealed that nearly all taxonomic descriptions on product labels were incorrect, likely stemming from the challenges associated with the precise identification of *Trichoderma* species (Dou et al., 2020). Among the ten analyzed isolates, six could not be accurately identified at the species level due to incomplete or ambiguous matches of either the TEF1, RPB2 and ITS sequences with known species. This uncertainty is compounded by ongoing taxonomic revisions of *Trichoderma*, largely attributed to advances in whole-genome sequencing and phylogenetic analyses (Schalamun and Schmoll, 2022). Three of the biological products assessed were labeled with the name *T. harzianum*, based on the misconception that it represents a single species. However, emerging research indicates that this taxon comprises multiple distinct species, complicating their precise characterization (Chaverri et al., 2015).

The specific properties of strains used as active ingredients can significantly influence consumer decision-making in relation to their application across various crops and environmental conditions. Although patent descriptions often provide detailed information about strains and their performance, such information was lacking for all analyzed biofertilizers. Furthermore, producer websites typically offered only generic descriptions of the beneficial role of *Trichoderma-*based biofertilizers without scientifically validated evidence. The lack of quality control measures for these products raises concerns about the proliferation of low-quality offerings in the marketplace. Especially, in developing countries, inadequate facilities for producing and storing high-quality inoculants frequently present a significant challenge (Herrmann and Lesueur, 2013). To tackle the observed problems, we recommend implementing a variety of targeted solutions. Firstly, regular monitoring of CFUs and other viability metrics for biofertilizers should be standard practice. Incorporating genetic tools such as MLST by sequencing specific loci (e.g., ITS, TEF1, and RPB2) can significantly enhance the accuracy of strain identification. In addition, the use of WGS could offer a means of monitoring genetic stability throughout the production process. The proposed roadmap for implementing advanced taxonomic verification methods should include controls to identify and manage genetic drift during culturing. Developing guidelines that incorporate the use of genomic tools for verifying the identity of microbial products will facilitate better regulatory acceptance. This requires collaboration with regulatory agencies to establish standard practices for strain verification using genomic data. Data on strain verification should be provided as clear labeling on products, detailing the genetic information of the strains used. Drawing on principles from frameworks such as ISO (International Organization for Standardization) and GMP (Good Manufacturing Practices), we propose a minimal set of regulatory standards or certification schemes for Trichoderma products. The outline of the proposed standards should include: (i) Strain Identity Verification through the use of genetic tools. This will confirm the identity of *Trichoderma* strains and prevent the use of misidentified products. (ii) *In vitro* and *In vivo* Efficacy Testing. As found in our study, isolates of *Trichoderma* show variation in terms of their invasion capacity, which could be included as labeling characteristics. (iii) Quality Control Procedures involving the implementation of stringent quality control measures for microbial formulations, including checks for viable cell counts, contamination levels, and stability under storage conditions. (iv) Safety Assessments by testing for pathogenicity and any potential adverse effects on humans, crops, and the environment. (v) Environmental Impact Studies through the evaluating the environmental persistence, colonization capacity of isolates and also investigate interactions of *Trichoderma* strains with plant root microbiomes to evaluate indirect plant health effects. (vi) Labeling Requirements, which mandate clear and comprehensive labeling that provides end users with critical information on the product’s formulation, application methods, storage recommendations, and safety precautions. Labels should also disclose strain identity and efficacy data supporting their claims. (vii) Training and Education Programs that establish mandatory training programs for both manufacturers and users to promote proper application methods. (viii) Post-Market Surveillance, which implements a system to continuously monitor the performance of Biological Control Agents (BCAs) in the field after their release. (ix) Standard Operating Procedures (SOPs), which involve developing clear SOPs that document best practices for the production, storage, and application of BCA. We believe that adopting these proposed regulatory standards will foster the development of reliable and effective biological products, thereby enhancing their acceptance and efficacy in sustainable agriculture. Comprehensive field trials are imperative to confirm laboratory findings and better understanding the drivers of *Trichoderma* efficacy. Future field trials should aim to correlate the antifungal activity of the isolates with field efficacy and incorporate secondary metabolite profiling to link chemical signatures with biocontrol activity. Employment of predictive modeling (e.g., using machine learning) to forecast efficacy based on genomic or metabolomic inputs could assist in rapid screening of future products. Additionally, field trials will be crucial in assessing the performance and persistence of selected *Trichoderma* strains under varying environmental conditions. Such trials can reveal how these strains behave in different agro-climatic contexts, providing insights essential for optimizing their use as biocontrol agents. Lastly, encouraging transparency and accessibility of research findings related to biocontrol of plant diseases will facilitate better consumer relations and pave the way for the continued success of these biotechnological innovations in agriculture. By addressing these challenges, the *Trichoderma* market can enhance the credibility of its products, fostering a safer and more reliable approach to biological control of plant pathogens.

## Data Availability

The datasets presented in this study can be found in online repositories. The names of the repository/repositories and accession number(s) can be found at: https://www.ncbi.nlm.nih.gov/genbank/, PRJNA1017172.
