# Supplementary material for: In-depth comparison of commercial Trichoderma-based products: integrative approaches to quantitative analysis, taxonomy and efficacy
Source: Front Microbiol. 2025 Sep 18;16:1646394. doi: 10.3389/fmicb.2025.1646394 (PMC12488588; doi:10.3389/fmicb.2025.1646394)

Confrontation (a) and magnification of the confrontation (b) between T-34 (upper hyphae) and *B. cinerea* (lower hyphae). *B. cinerea* was fully overgrown by T-34. Images were taken after three weeks of growth at 25 °C ± 1.

a

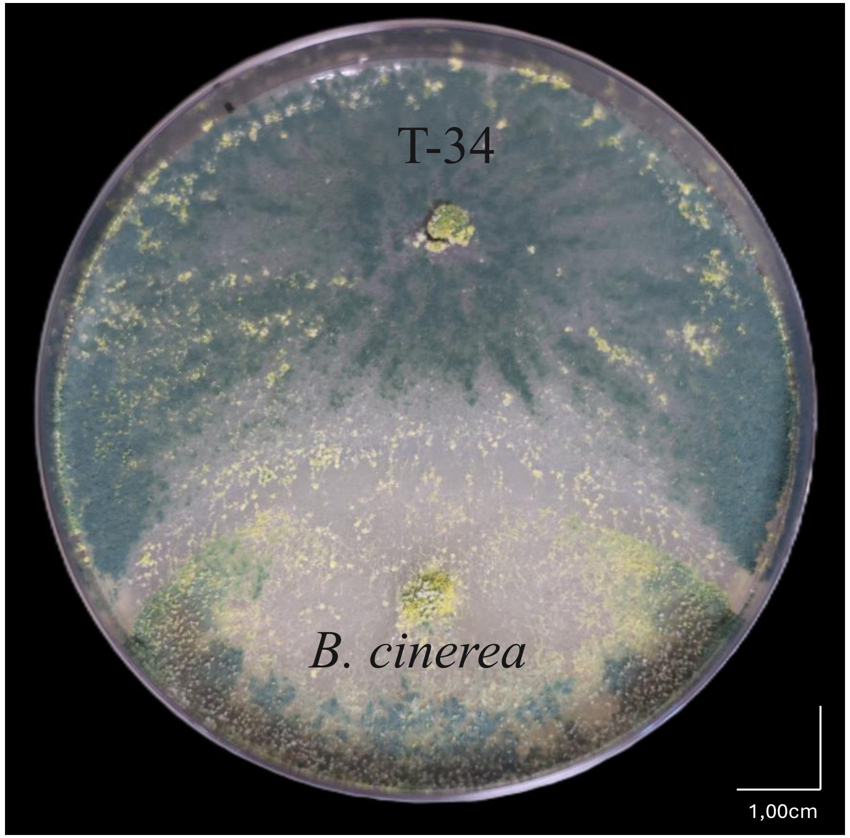

b

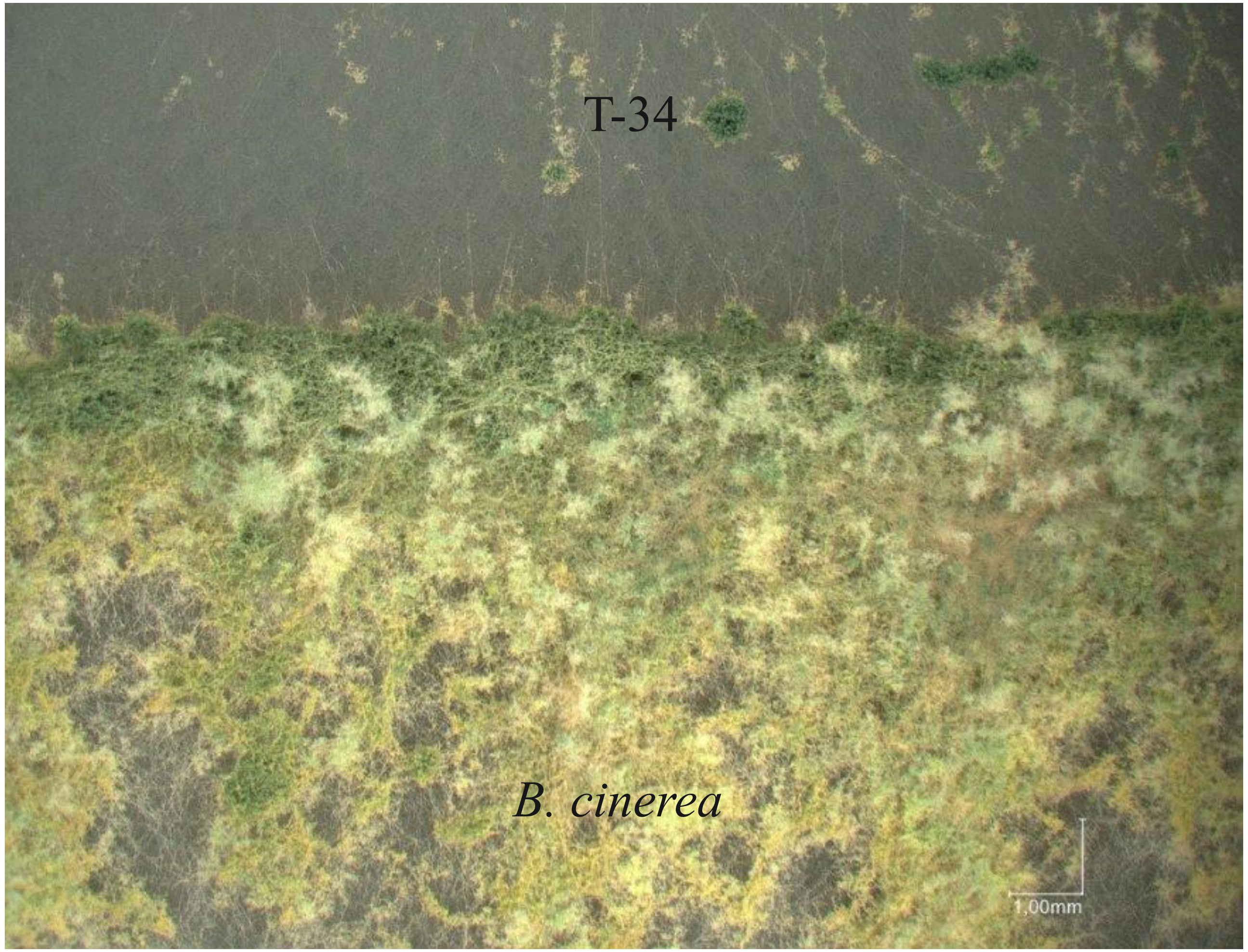

Confrontation (a) and magnification of the confrontation (b) between T-34 (upper hyphae) and *F. graminearum* (lower hyphae). *F. graminearum* was fully overgrown by T-34. Images were taken after three weeks of growth at 25 °C ± 1.

a

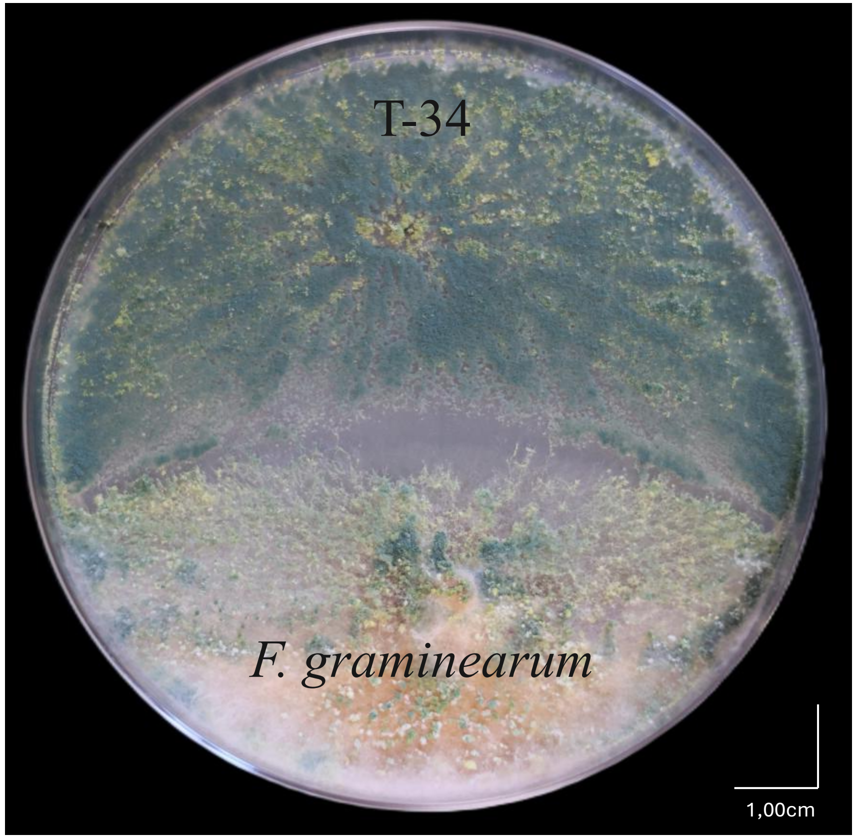

b

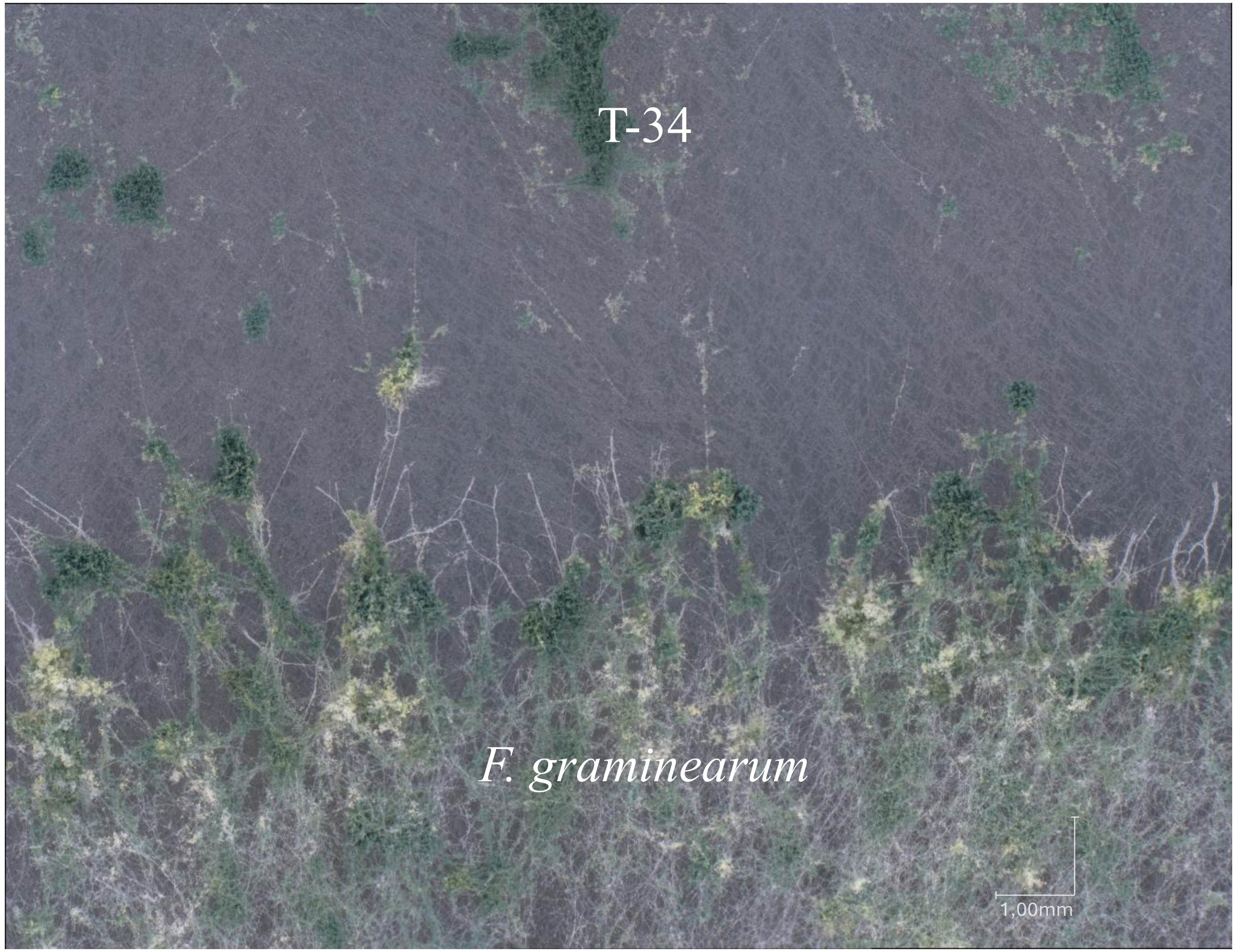

Confrontation (a) and magnification of the confrontation (b) between T-34 (upper hyphae) and *S. sclerotiorum* (lower hyphae). *S. sclerotiorum* was fully overgrown by T-34. Images were taken after three weeks of growth at 25 °C ± 1.

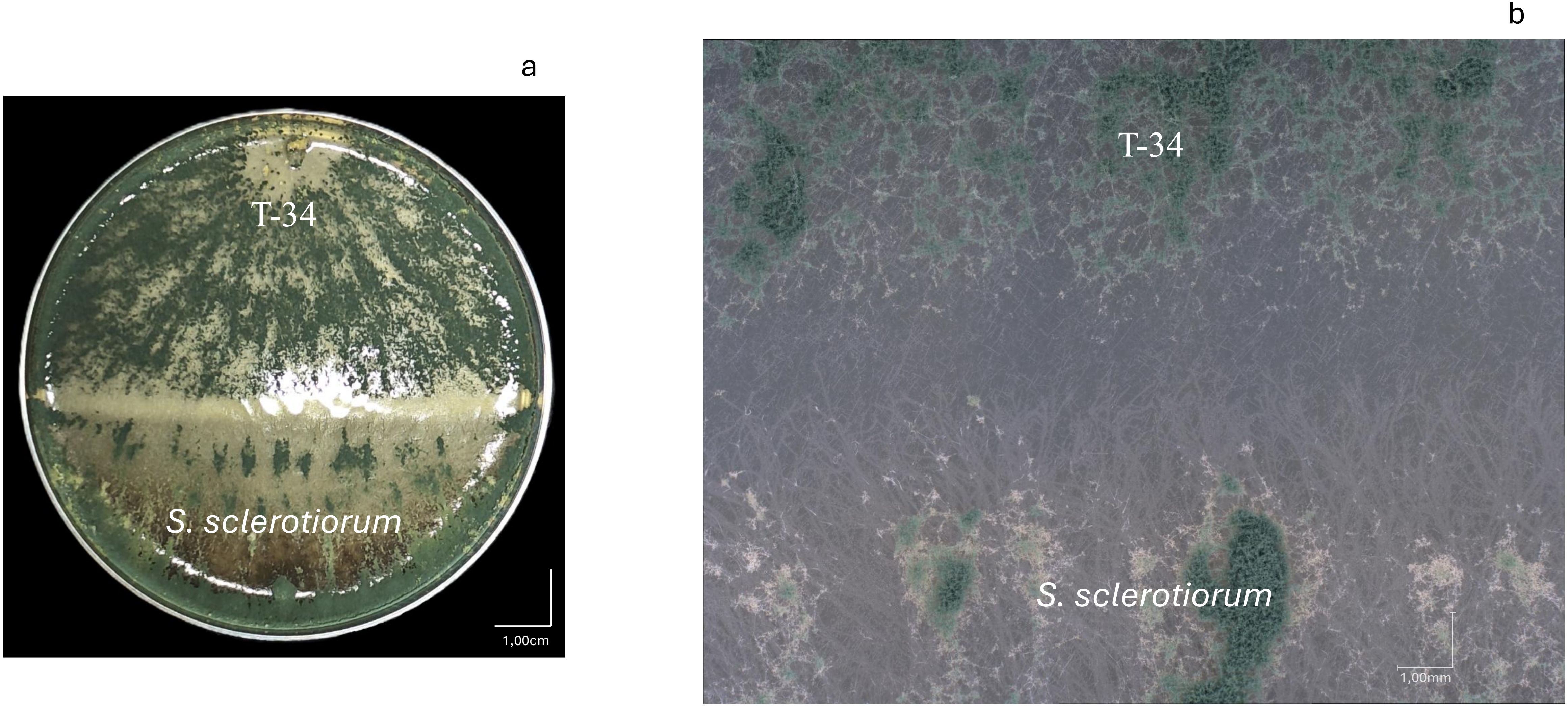

Confrontation (a) and magnification of the confrontation (b) between SC1 (upper hyphae) and *B. cinerea* (lower hyphae). *B. cinerea* was fully overgrown by SC1. Images were taken after three weeks of growth at 25 °C ± 1.

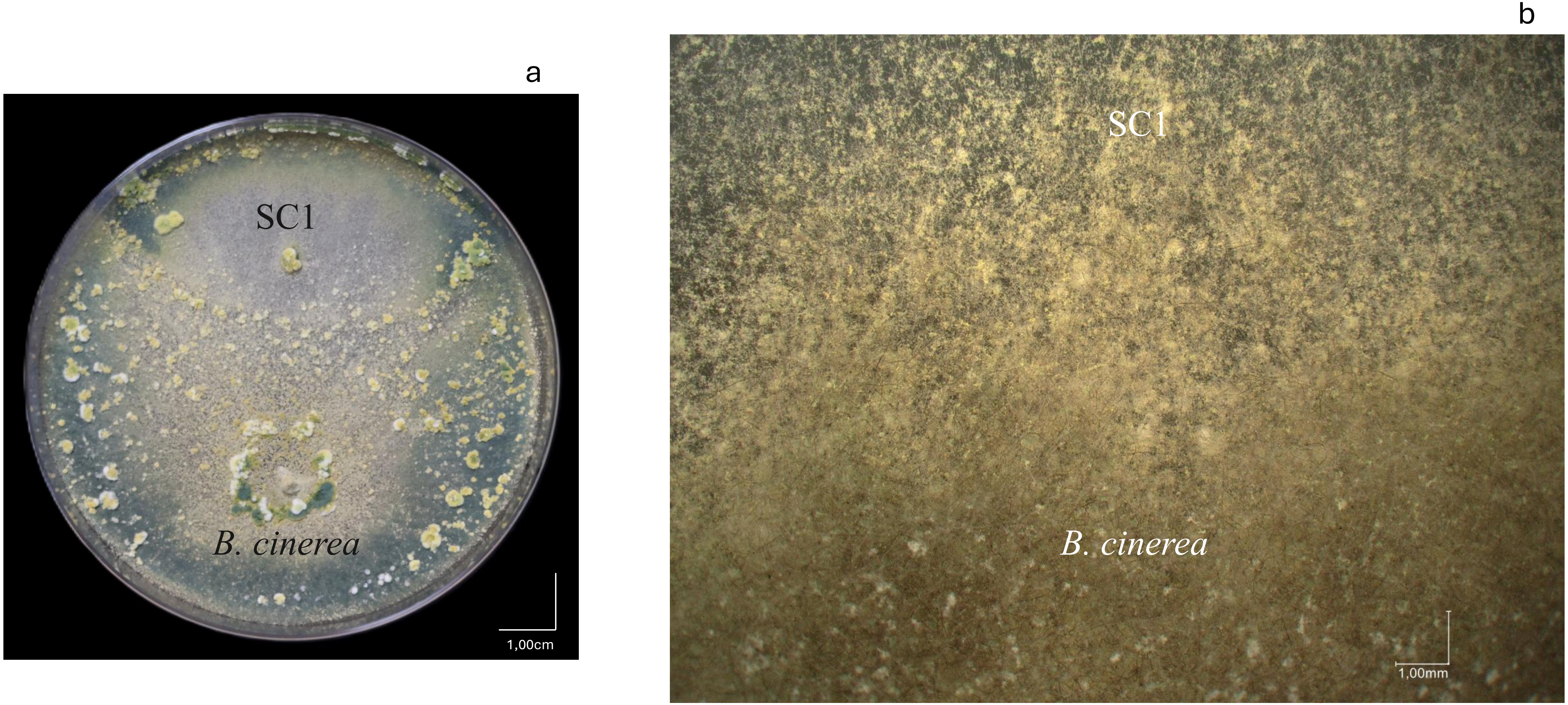

Confrontation (a) and magnification of the confrontation (b) between SC1 (upper hyphae) and *F. graminearum* (lower hyphae). *F. graminearum* was fully overgrown by SC1. Images were taken after three weeks of growth at 25 °C ± 1.

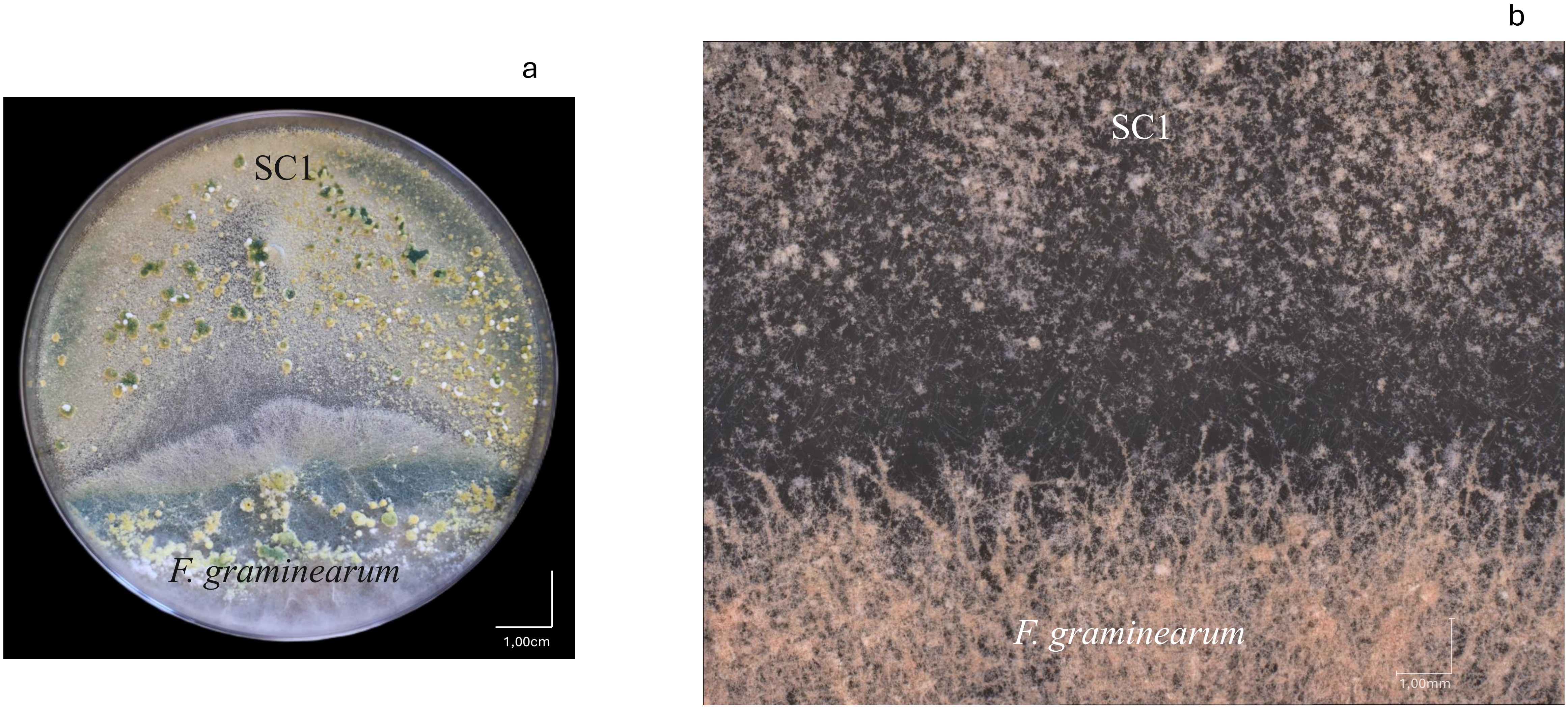

Confrontation (a) and magnification of the confrontation (b) between SC1 (upper hyphae) and *S. sclerotiorum* (lower hyphae). *S. sclerotiorum* was fully overgrown by SC1. Images were taken after three weeks of growth at 25 °C ± 1.

a

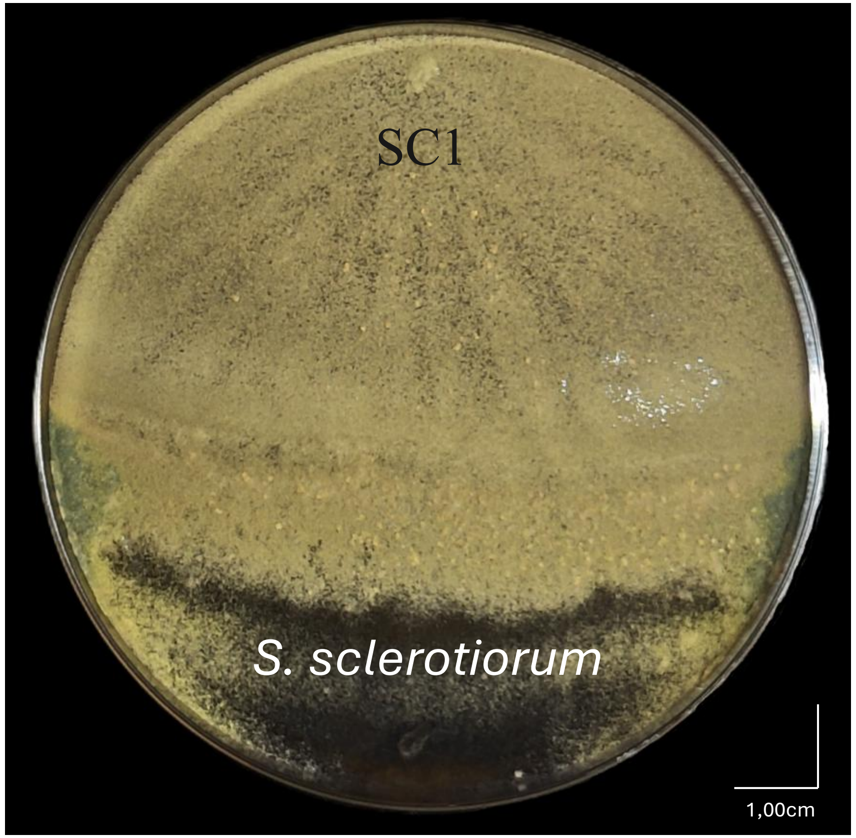

b

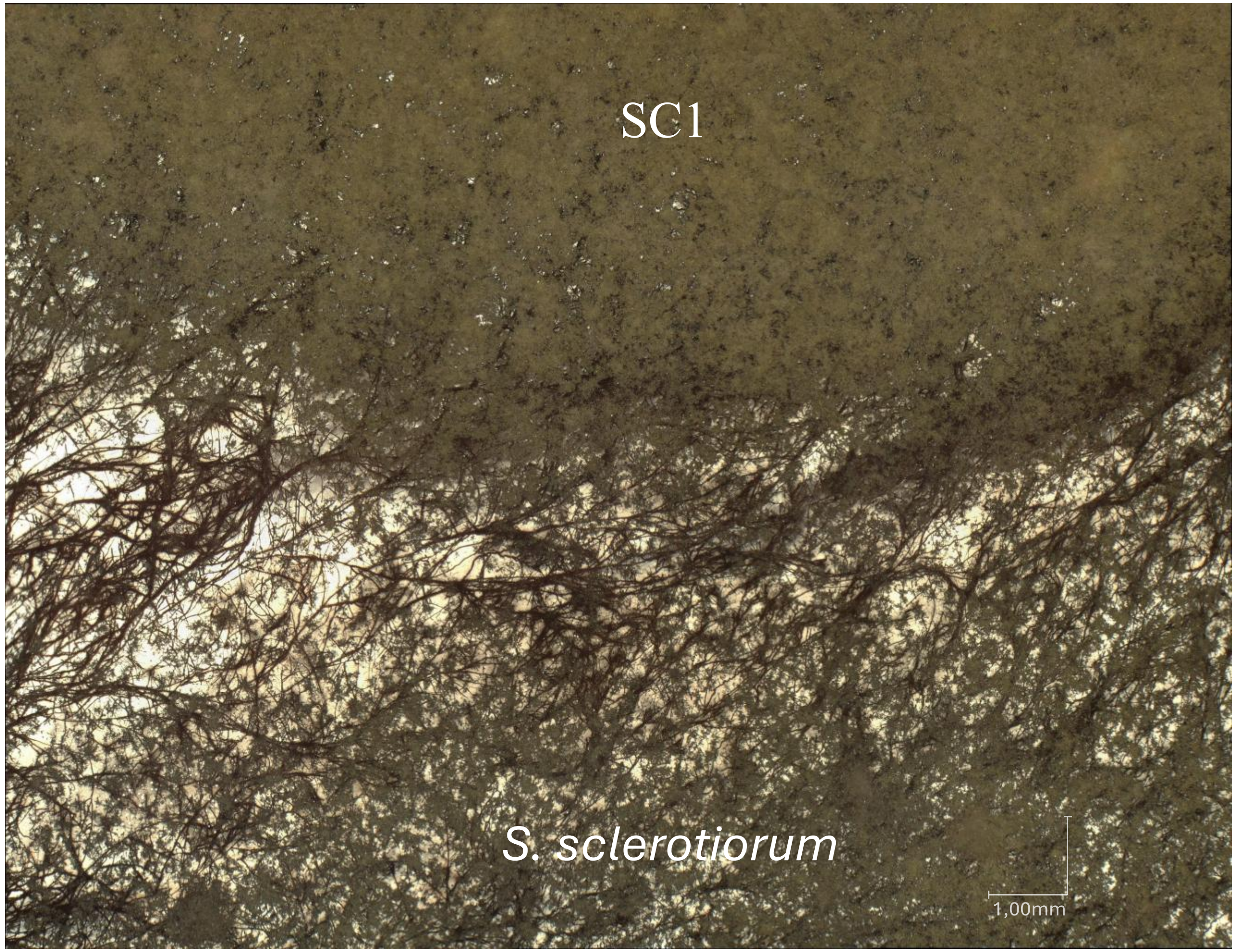

Confrontation (a) and magnification of the confrontation (b) between T-22 (upper hyphae) and *B. cinerea* (lower hyphae). *B. cinerea* was fully overgrown by T-22. Images were taken after three weeks of growth at 25 °C ± 1.

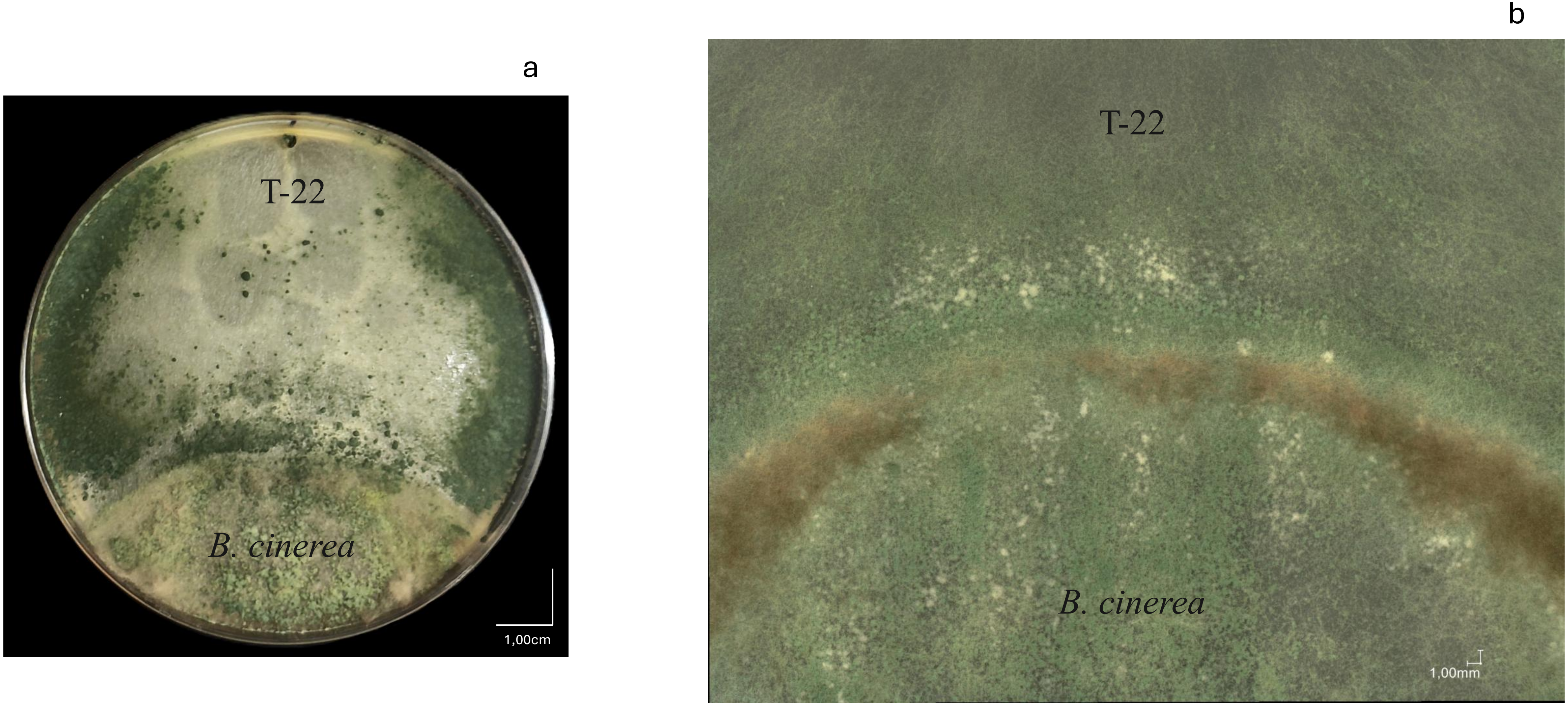

Confrontation (a) and magnification of the confrontation (b) between T-22 (upper hyphae) and *F. gramiearum* (lower hyphae). *F. graminearum* was not overgrown by T-22. Images were taken after three weeks of growth at 25 °C ± 1.

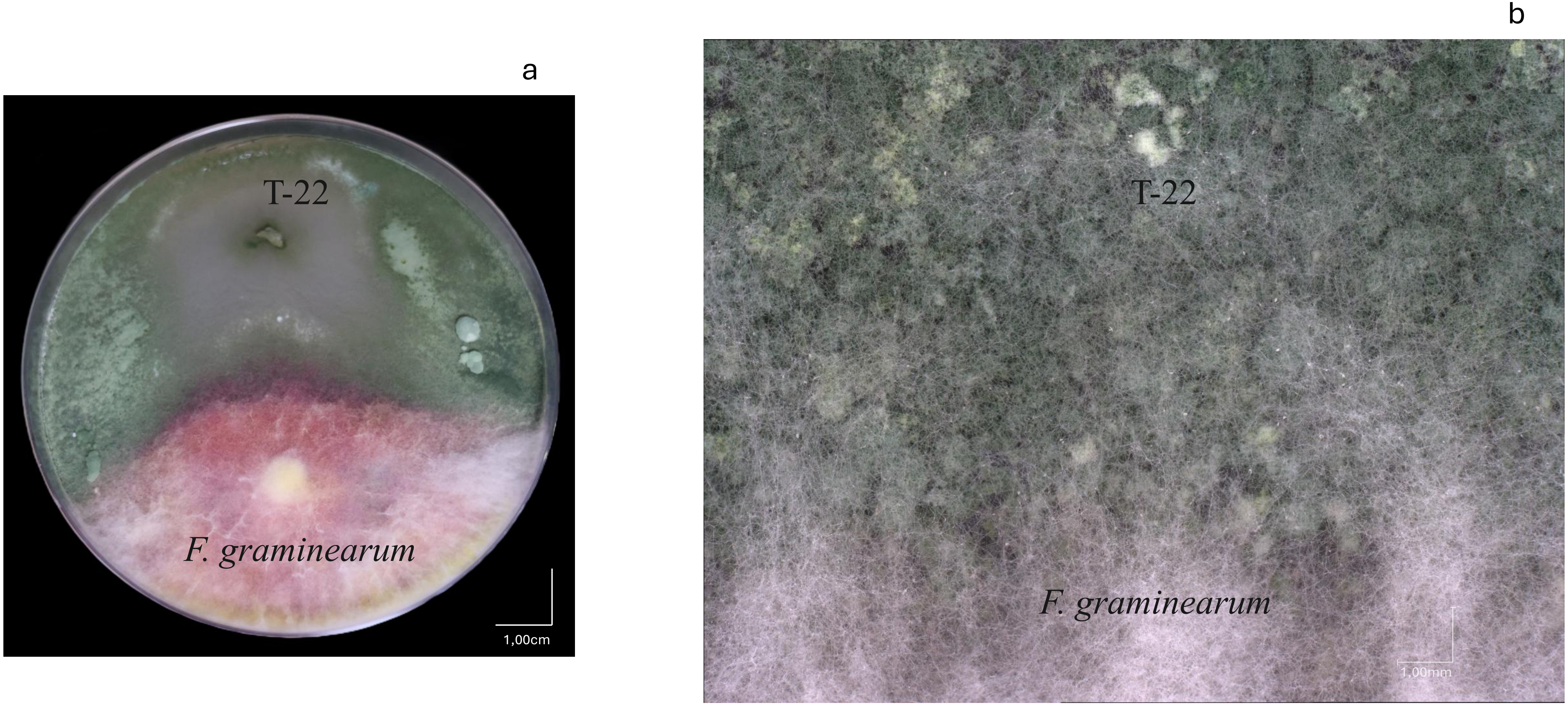

Confrontation (a) and magnification of the confrontation (b) between T-22 (upper hyphae) and *S. sclerotiorum* (lower hyphae). *S. sclerotiorum* was fully overgrown by T-22. Images were taken after three weeks of growth at 25 °C ± 1.

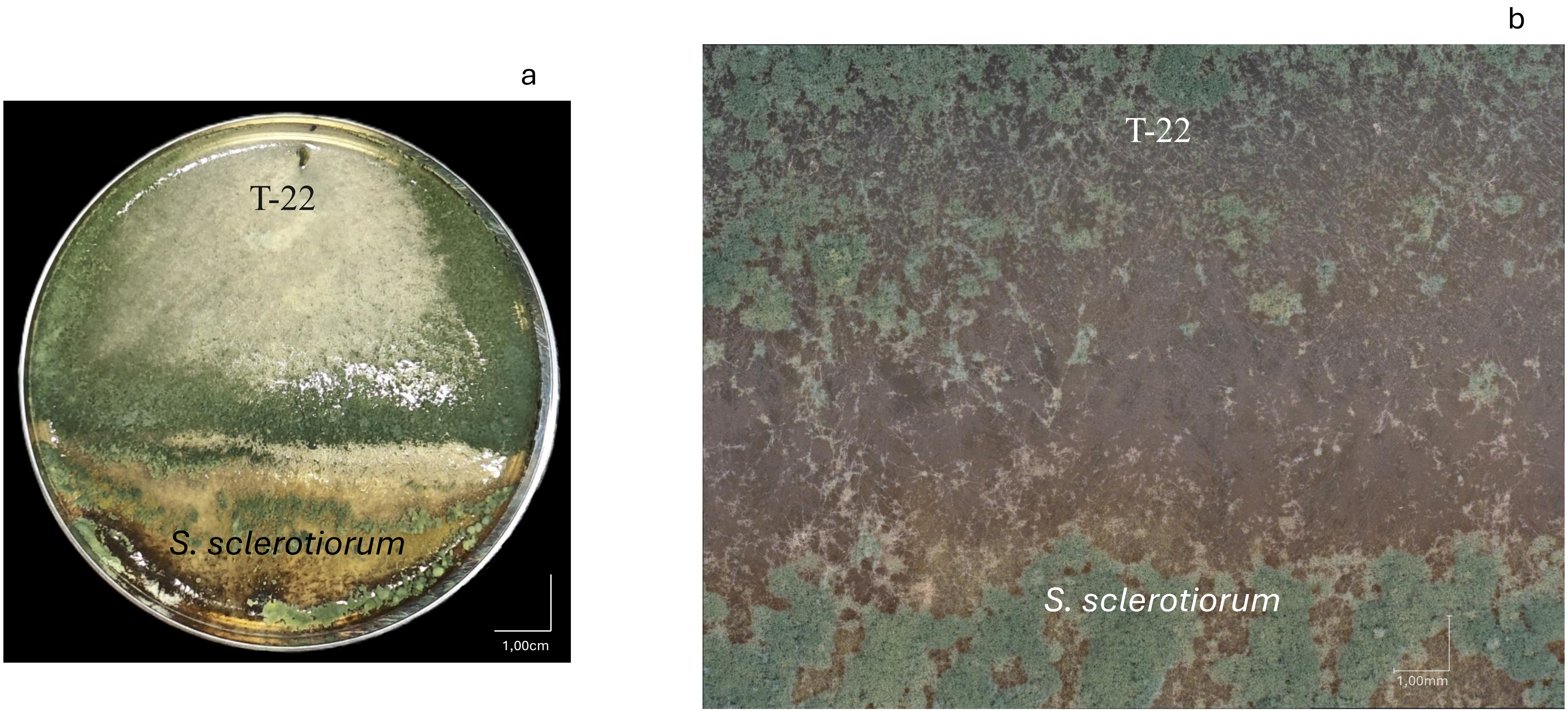

Confrontation (a) and magnification of the confrontation (b) between ICC 012 (upper hyphae) and *B. cinerea*(lower hyphae). *B. cinerea* was fully overgrown by ICC 012. Images were taken after three weeks of growth at 25 °C ± 1.

a

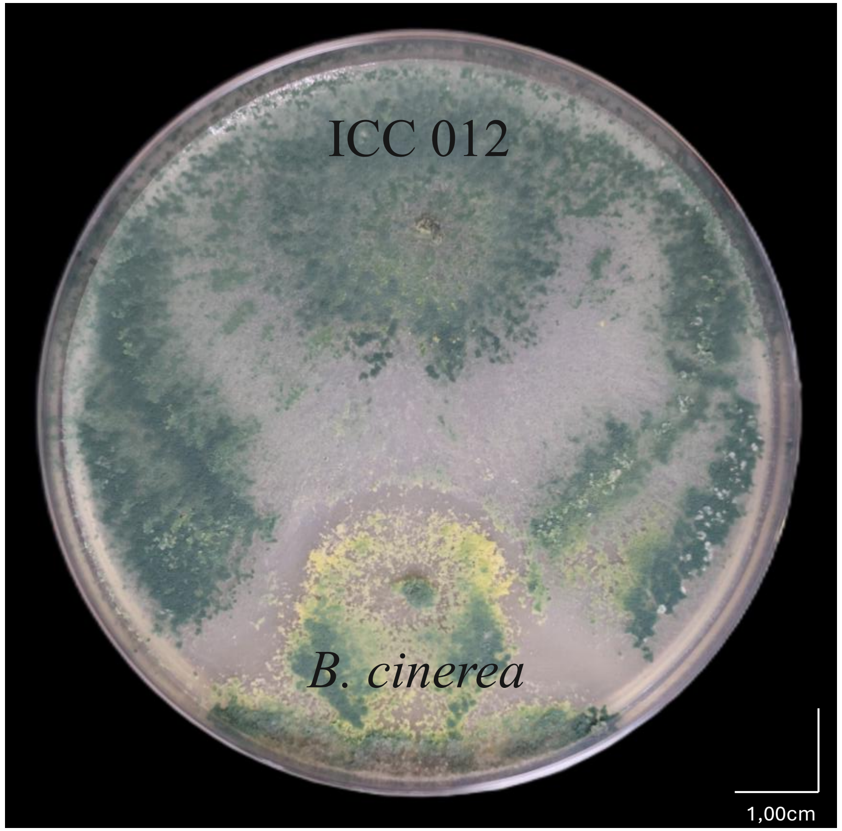

b

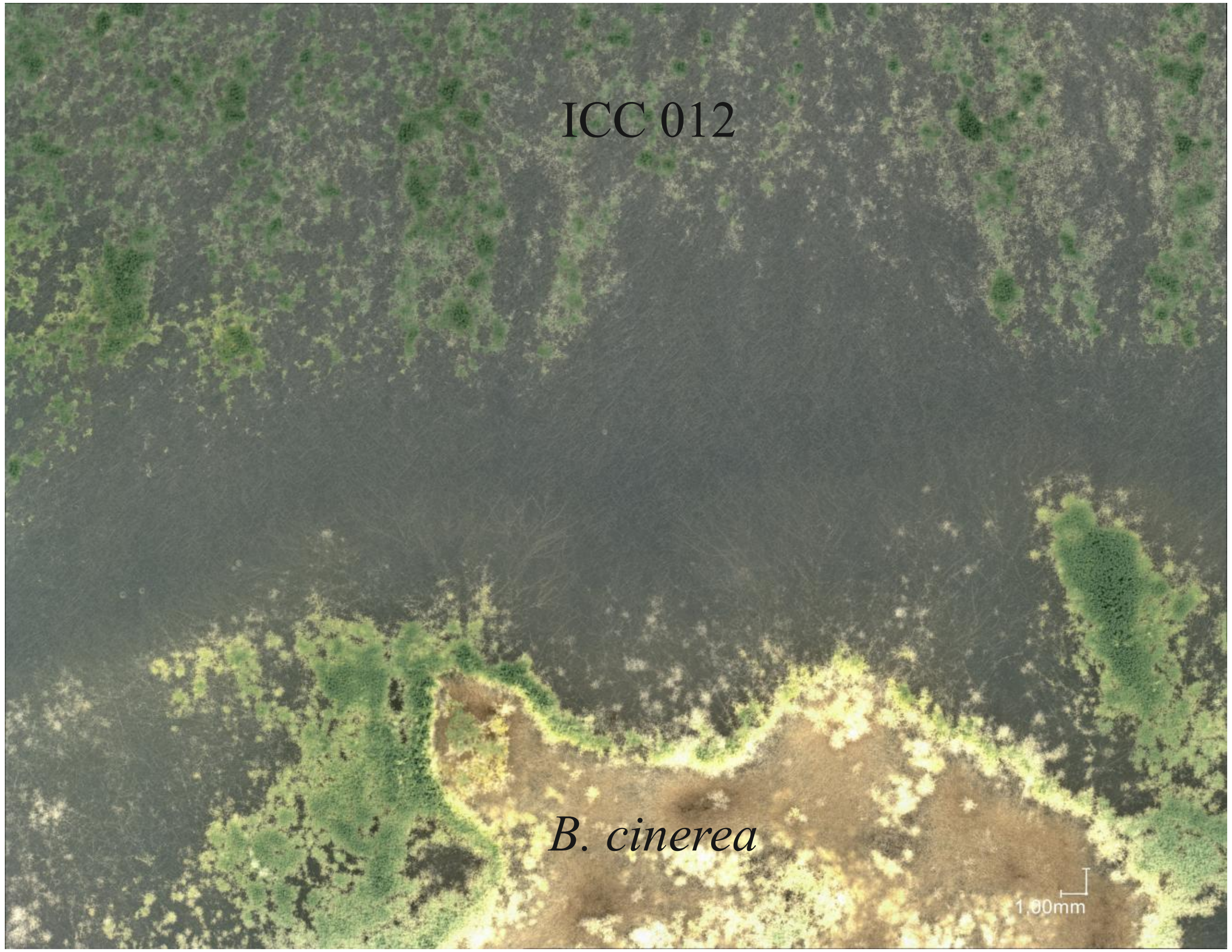

Confrontation (a) and magnification of the confrontation (b) between ICC 012 (upper hyphae) and *F. graminearum* lower hyphae). *F. graminearum* was fully overgrown by ICC 012. Images were taken after three weeks of growth at 25 °C ± 1.

a

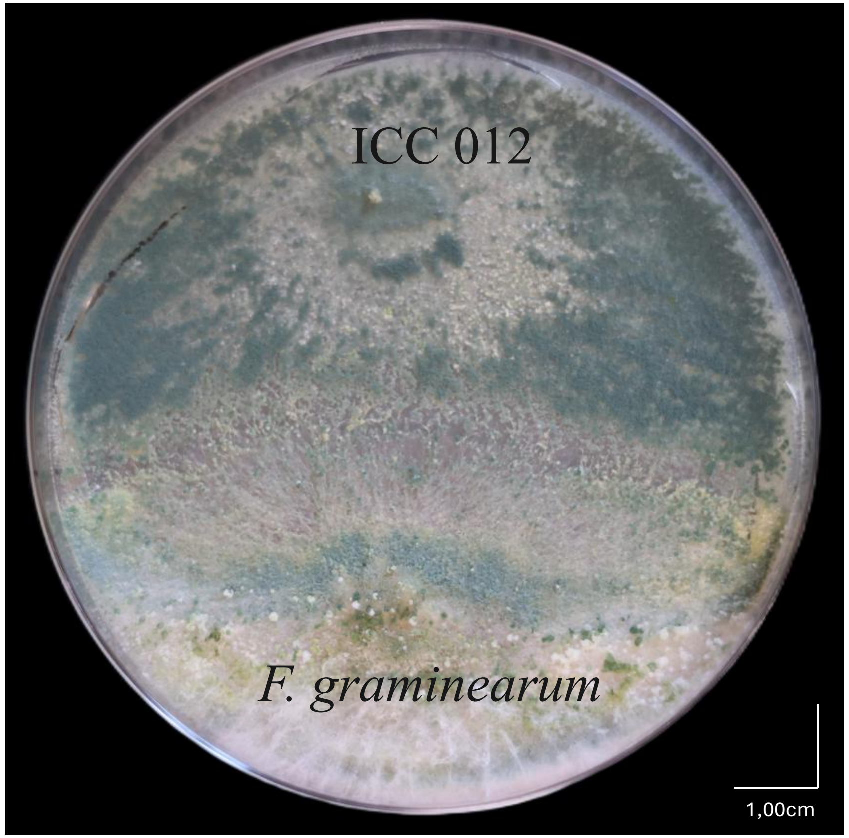

b

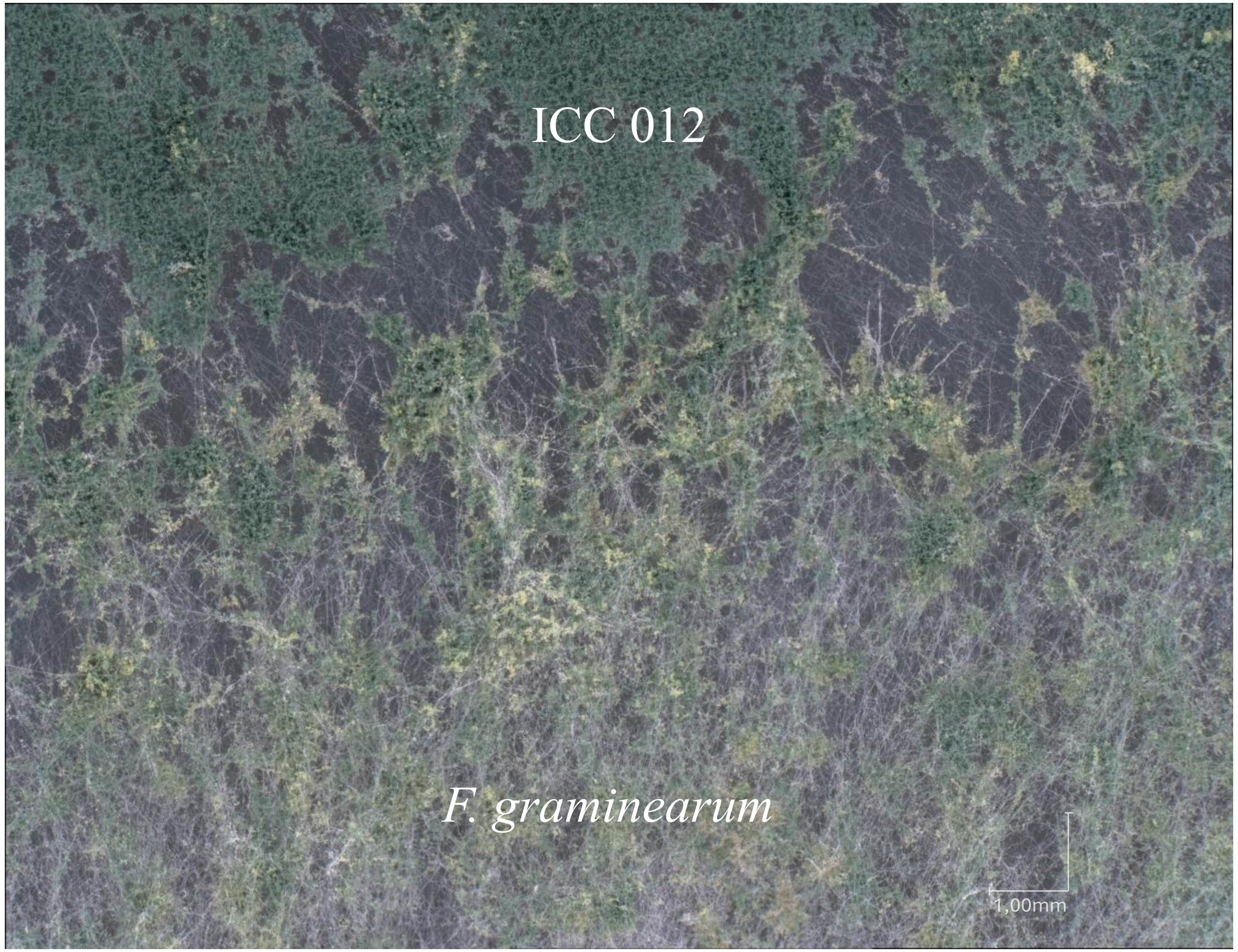

Confrontation (a) and magnification of the confrontation (b) between ICC 012 (upper hyphae) and *S. sclerotiorum* lower hyphae). *S. sclerotiorum* was fully overgrown by ICC 012. Images were taken after three weeks of growth at 25 °C ± 1.

a

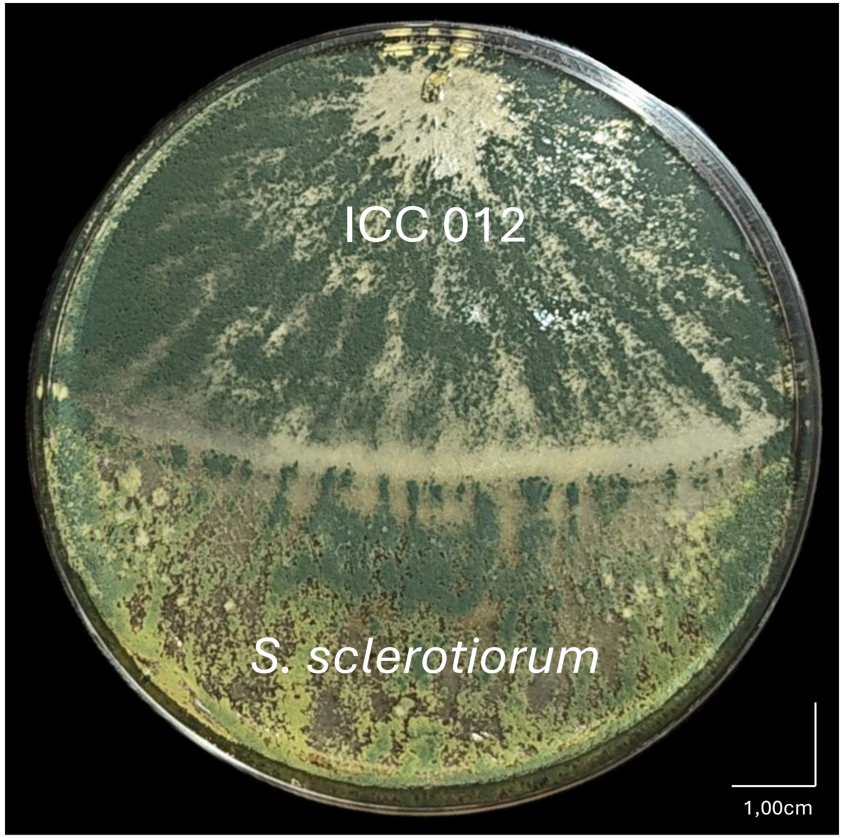

b

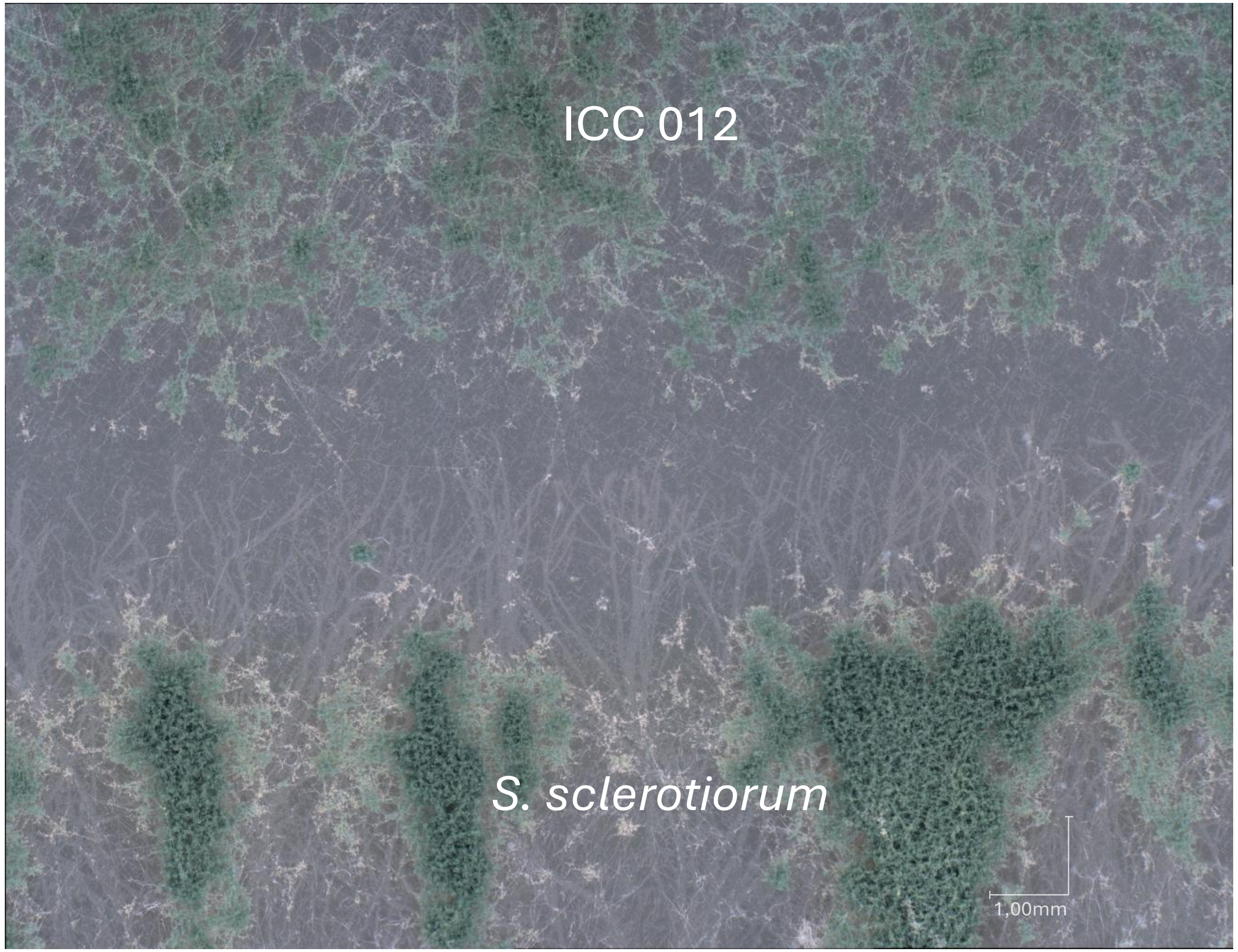

Confrontation (a) and magnification of the confrontation (b) between Tf1 (upper hyphae) and *B. cinerea* (lower hyphae). *B. cinerea* was weakly overgrown by Tf1. Images were taken after three weeks of growth at 25 °C ± 1.

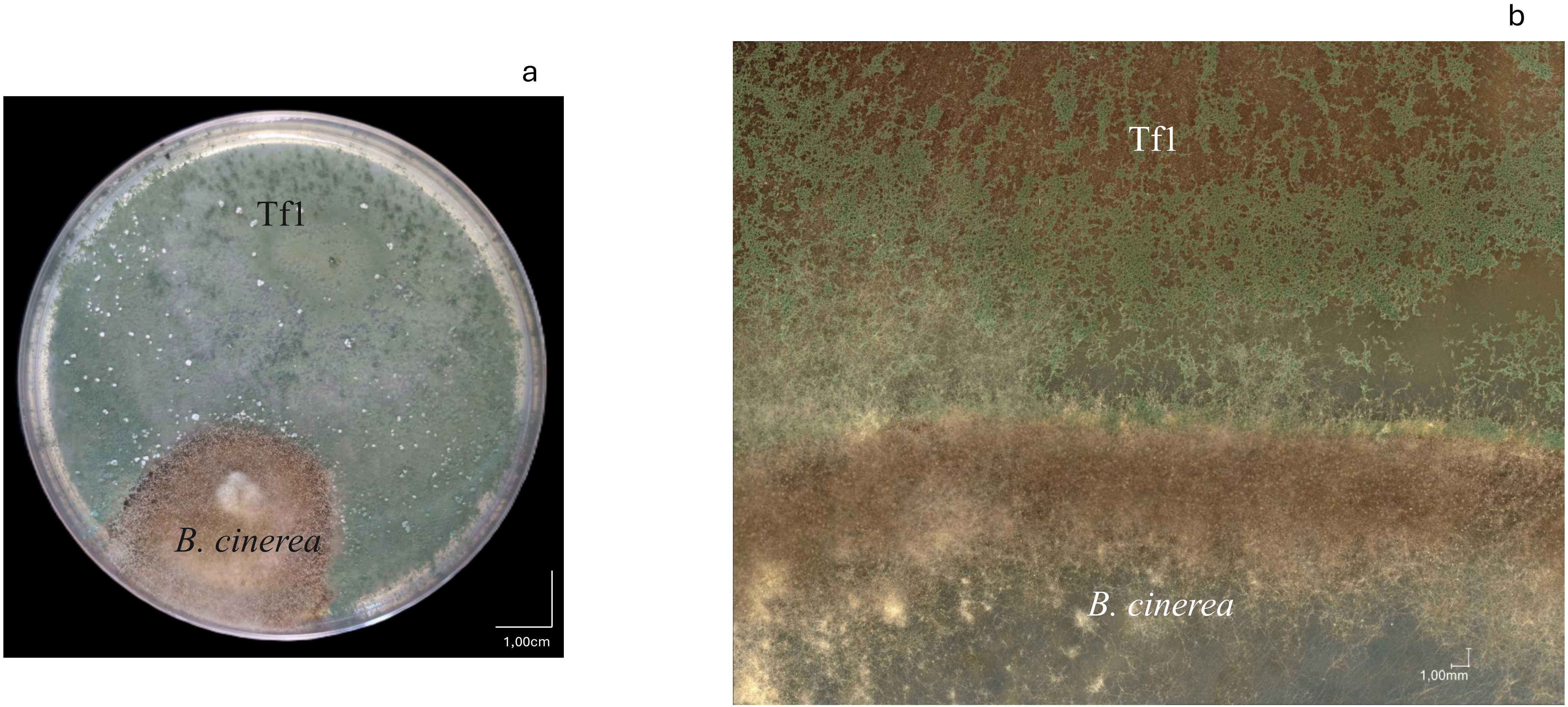

Confrontation (a) and magnification of the confrontation (b) between Tf1 (upper hyphae) and *F. graminearum* (lower hyphae). *F. graminearum* was weakly overgrown by Tf1. Images were taken after three weeks of growth at 25 °C ± 1.

a

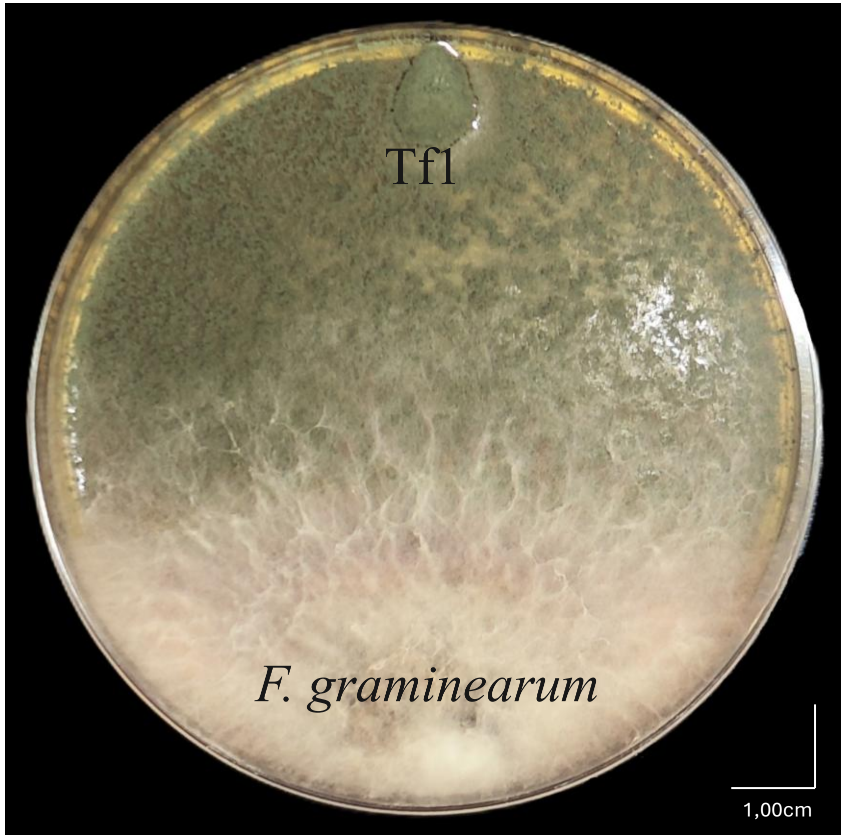

b

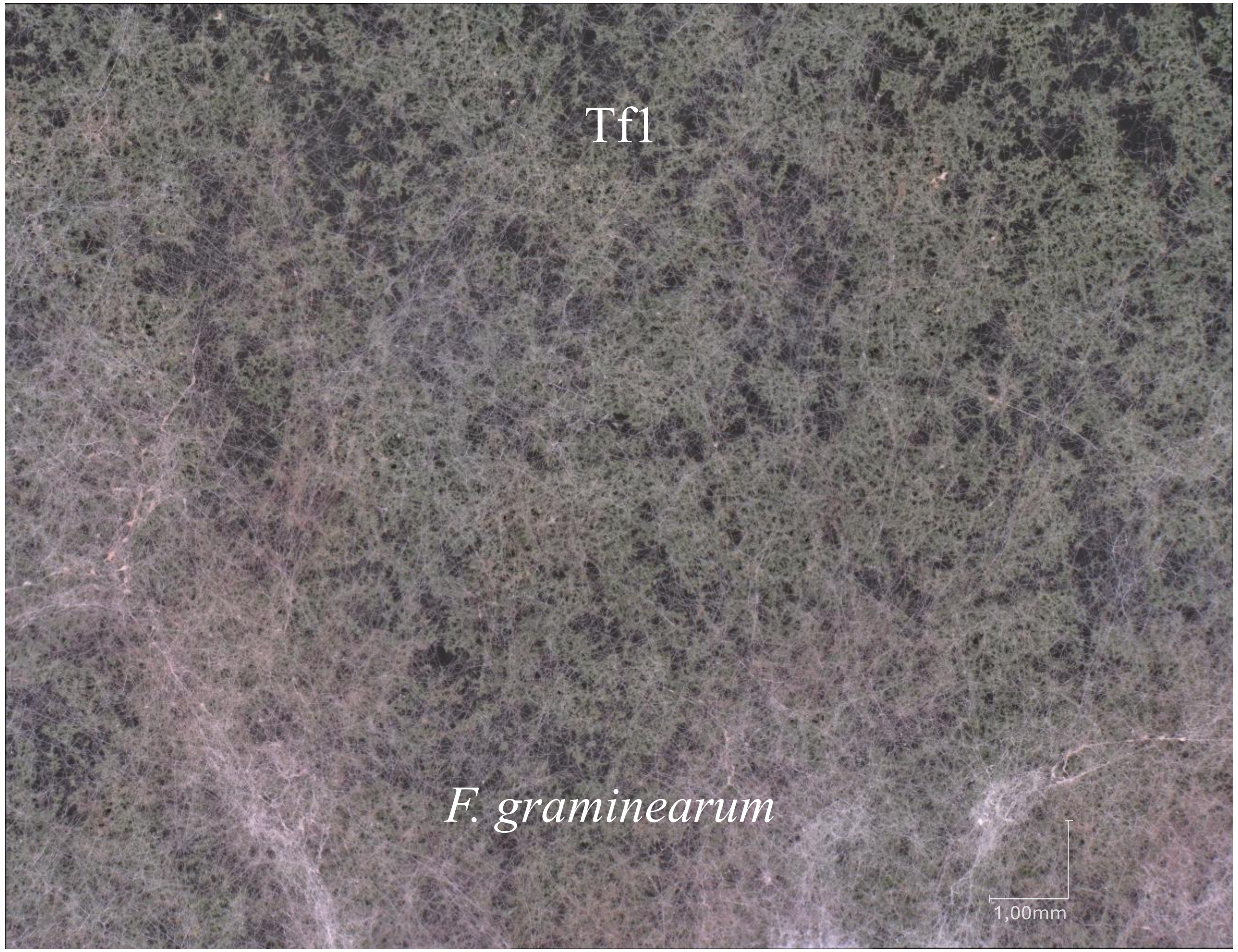

Confrontation (a) and magnification of the confrontation (b) between Tf1 (upper hyphae) and *S. sclerotiorum* (lower hyphae). *S. sclerotiorum* was weakly overgrown by Tf1. Images were taken after three weeks of growth at 25 °C ± 1.

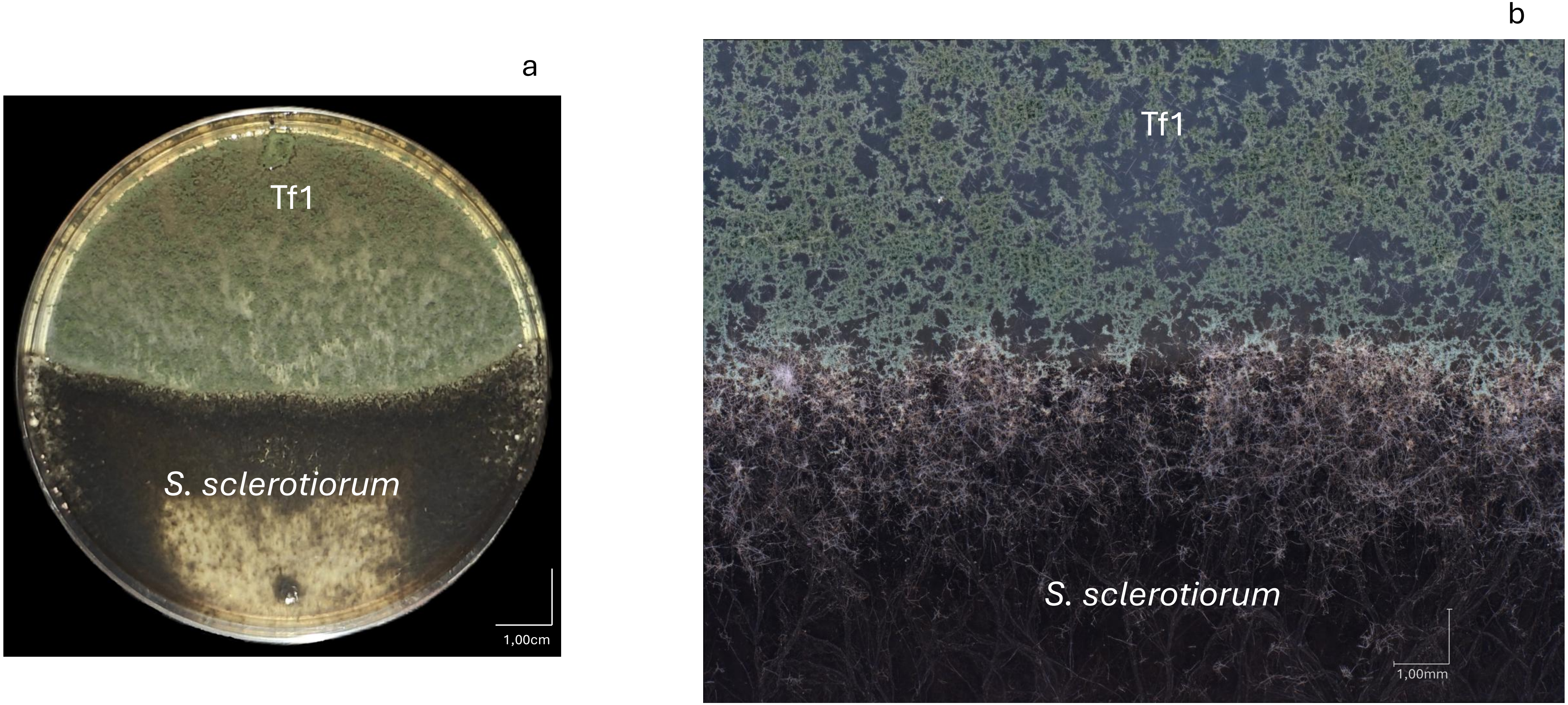

Confrontation (a) and magnification of the confrontation (b) between Cys1 (upper hyphae) and *B. cinerea* (lower hyphae). *B. cinerea* was fully overgrown by Cys1. Images were taken after three weeks of growth at 25 °C ± 1.

a

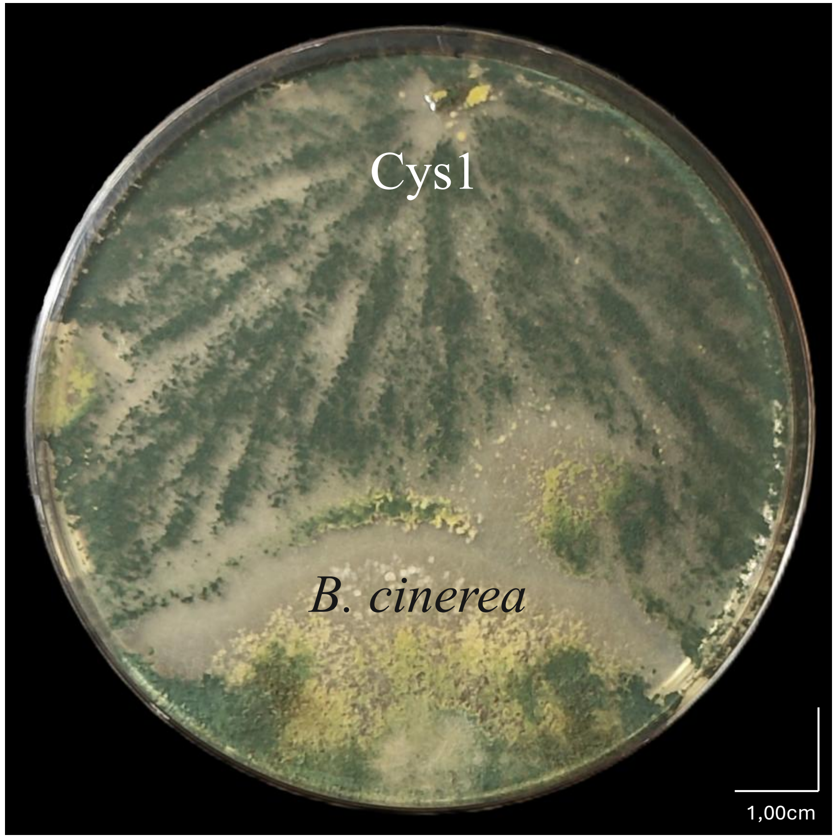

b

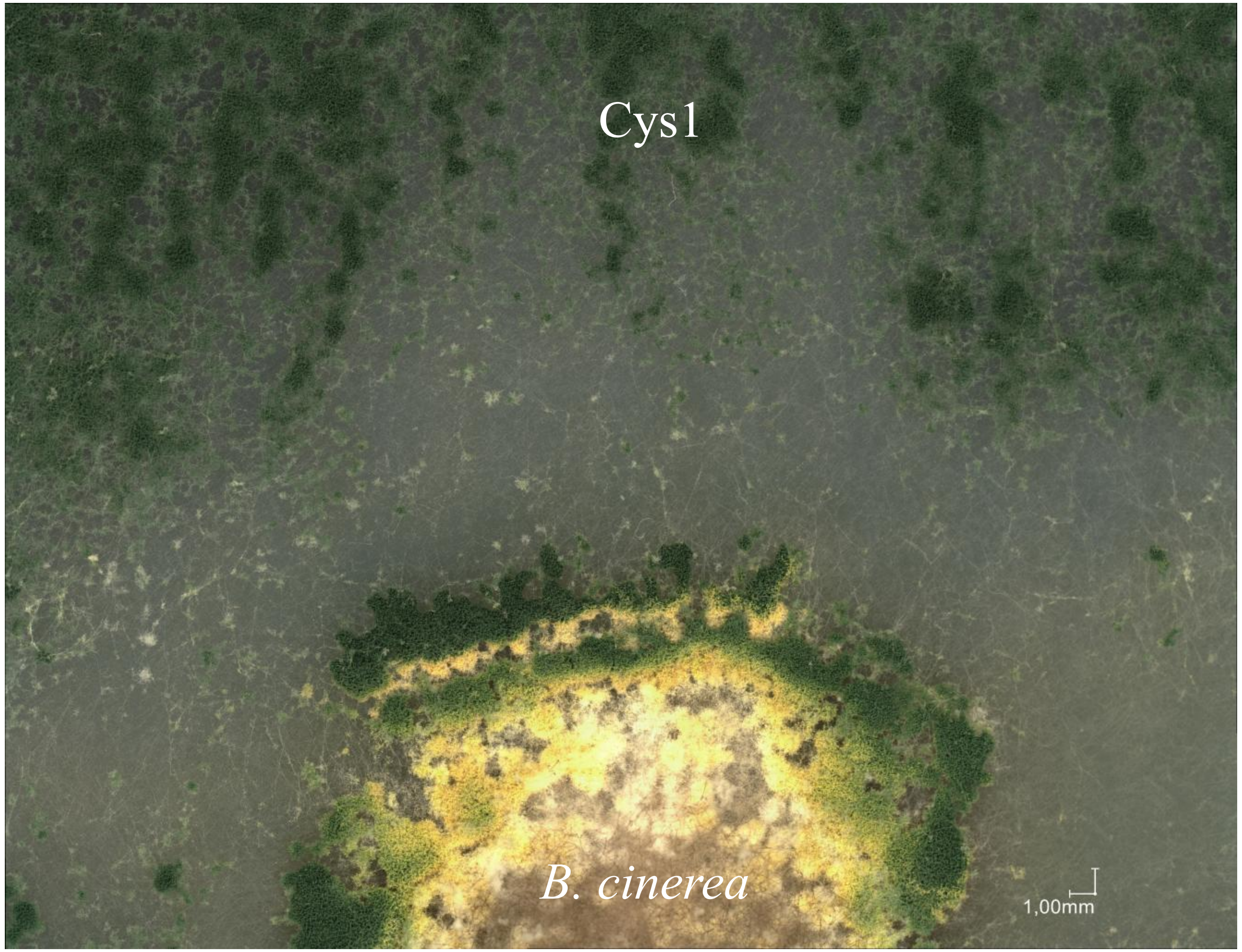

Confrontation (a) and magnification of the confrontation (b) between Cys1 (upper hyphae) and *F. graminearum* (lower hyphae). *F. graminearum* was partially overgrown by Cys1. Images were taken after three weeks of growth at 25 °C ± 1.

a

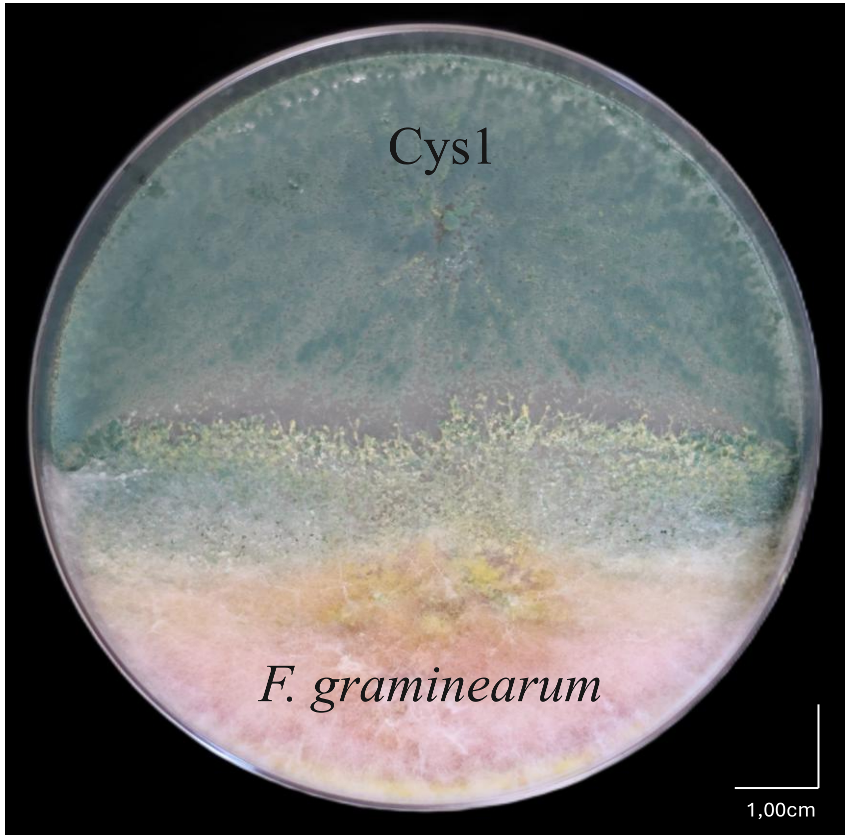

b

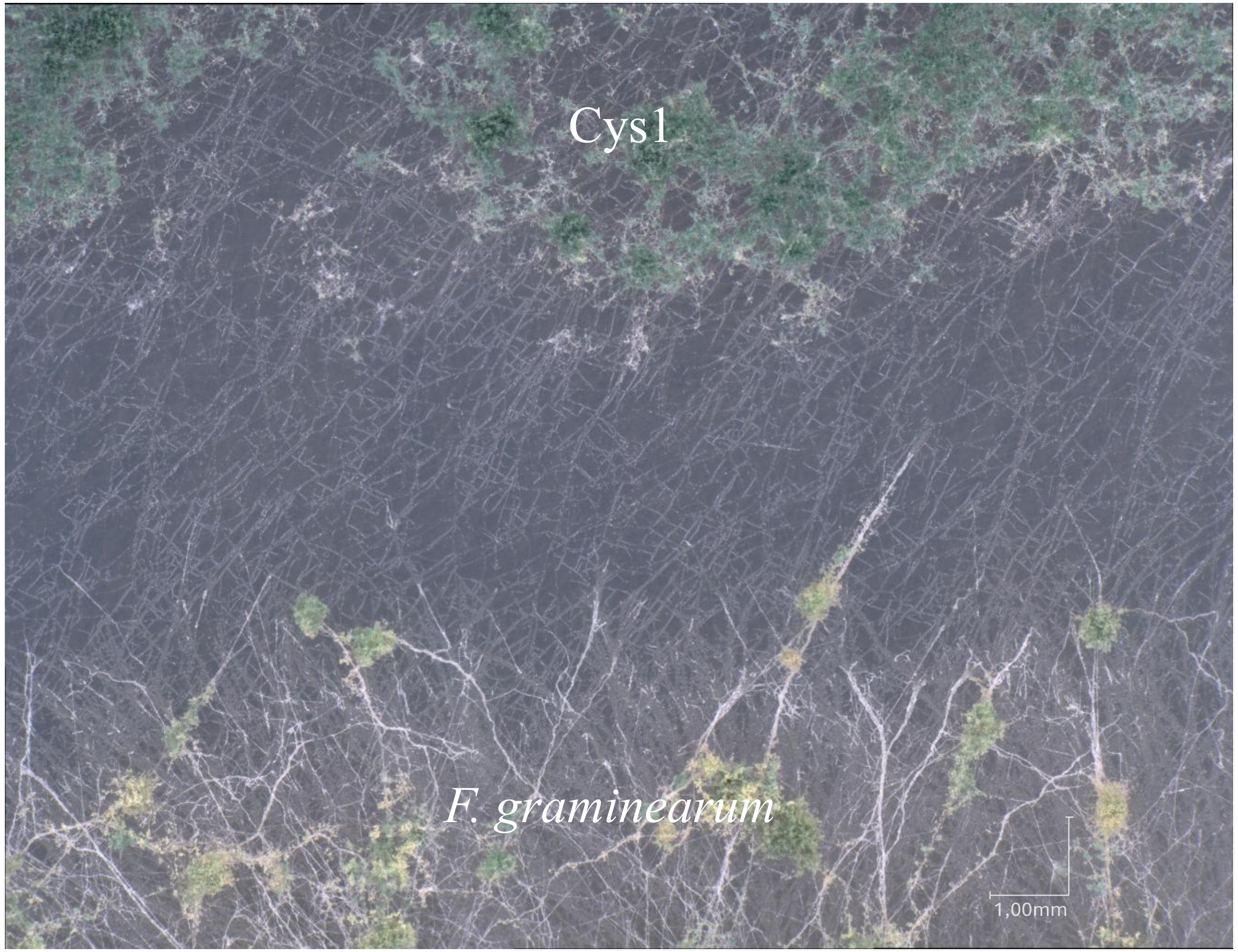

Confrontation (a) and magnification of the confrontation (b) between Cys1 (upper hyphae) and *S. sclerotiorum* (lower hyphae). *S. sclerotiorum* was fully overgrown by Cys1. Images were taken after three weeks of growth at 25 °C ± 1.

a

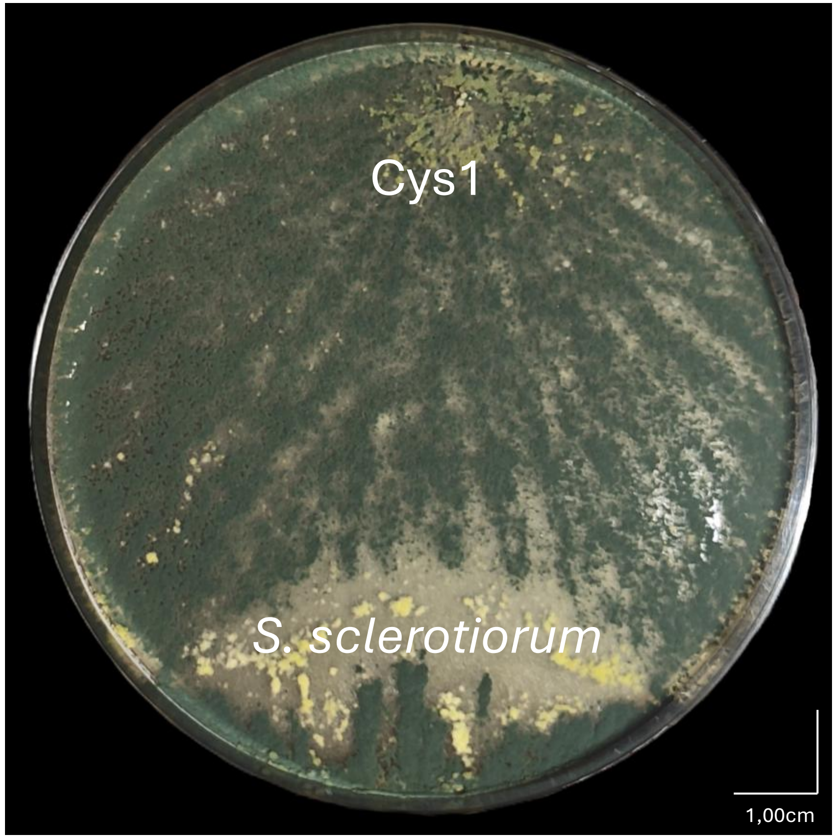

b

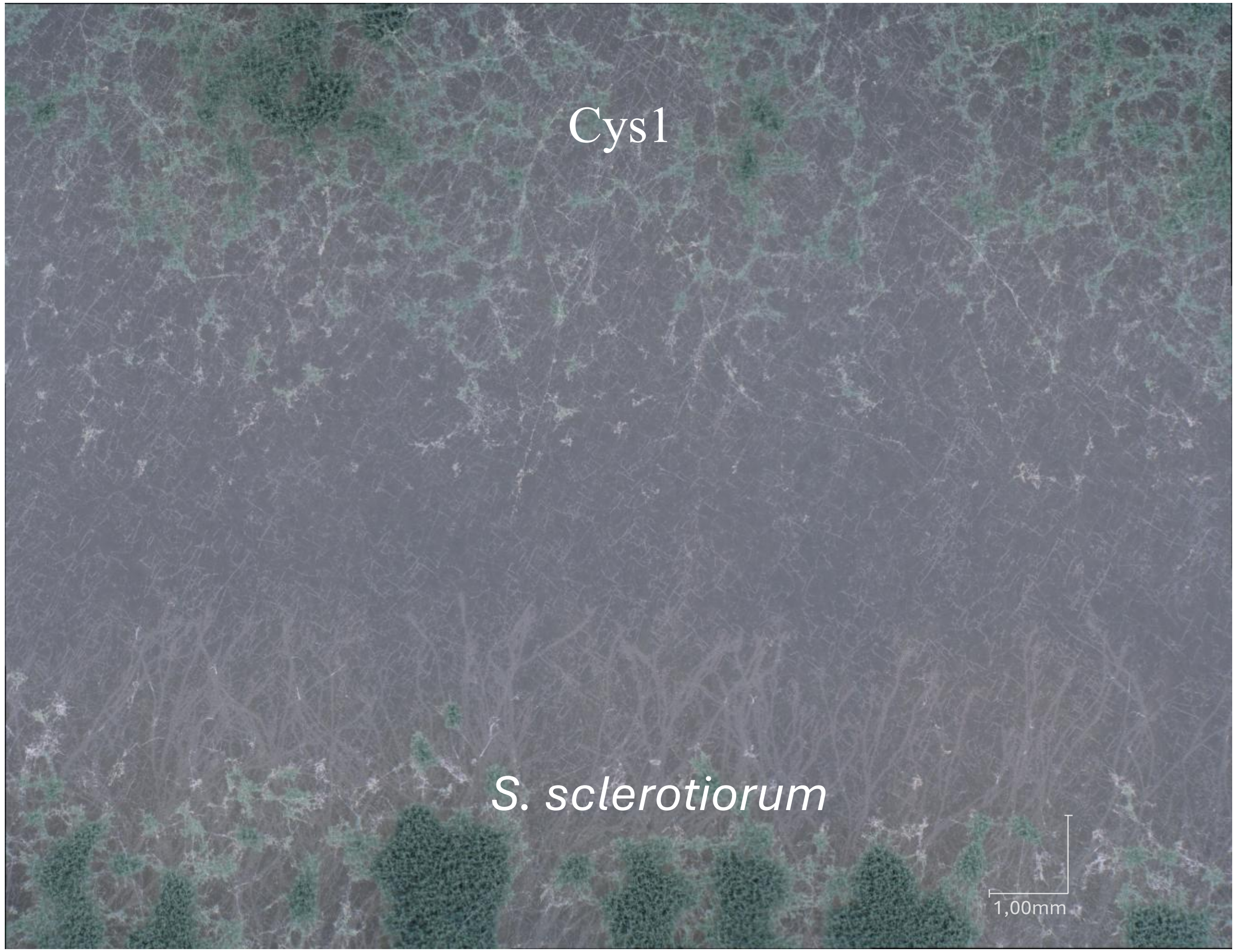

Confrontation (a) and magnification of the confrontation (b) between Cys2 (upper hyphae) and *B. cinerea* (lower hyphae). *B. cinerea* was not overgrown by Cys2. Images were taken after three weeks of growth at 25 °C ± 1.

a

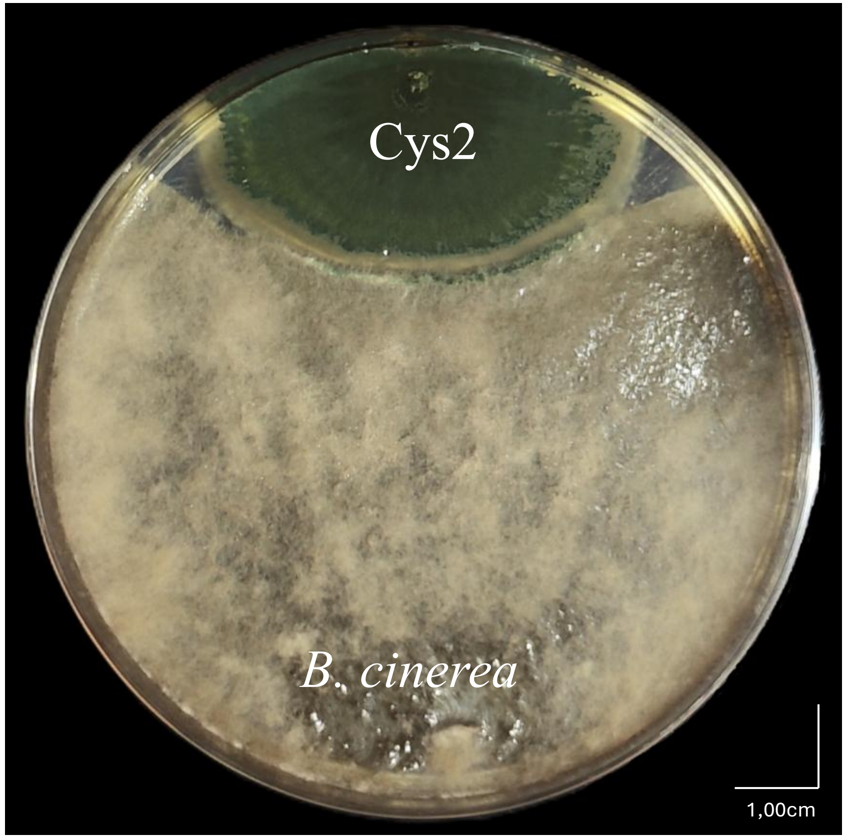

b

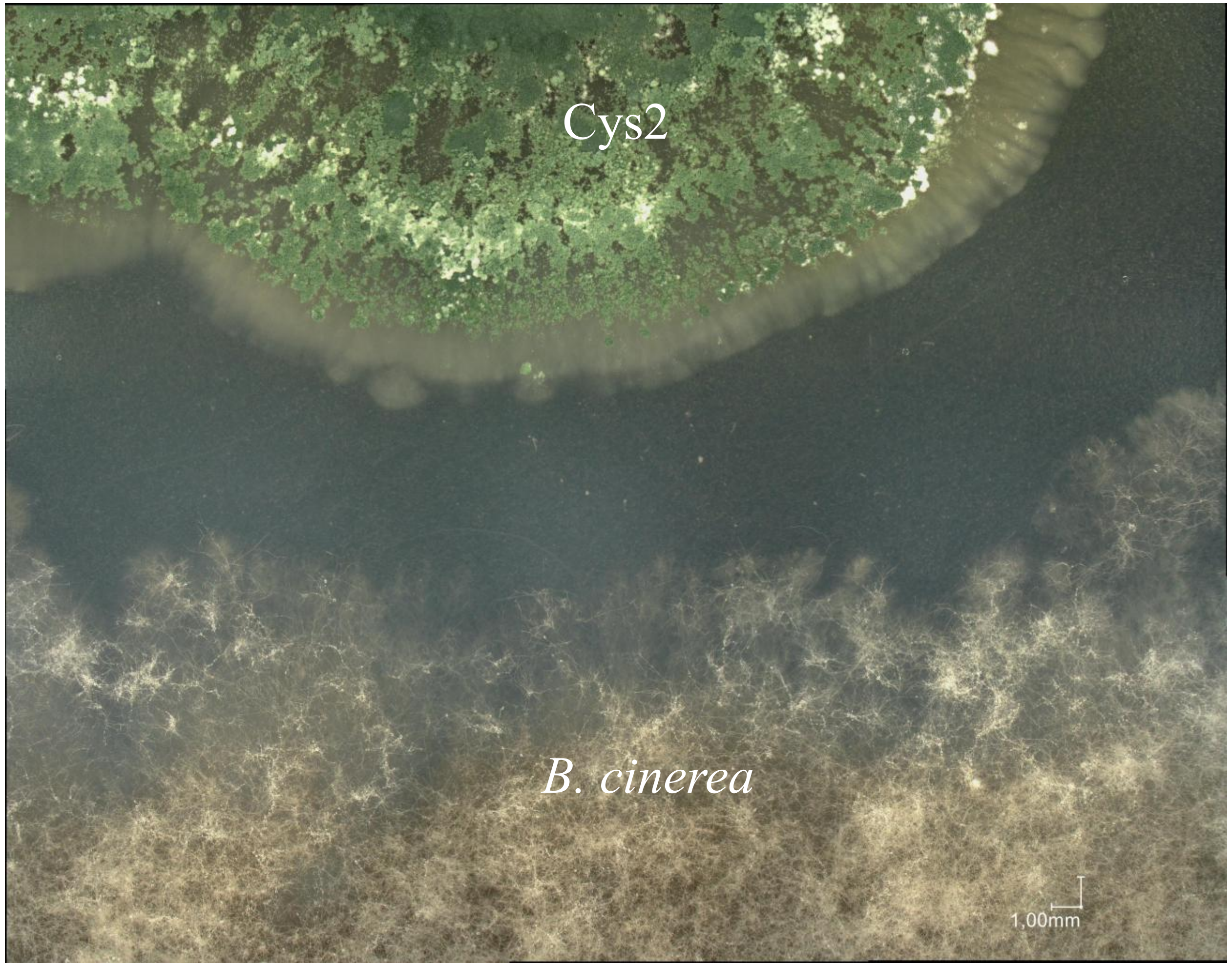

Confrontation (a) and magnification of the confrontation (b) between Cys2 (upper hyphae) and *F. graminearum* (lower hyphae). *F. graminearum* was not overgrown by Cys2. Images were taken after three weeks of growth at 25 °C ± 1.

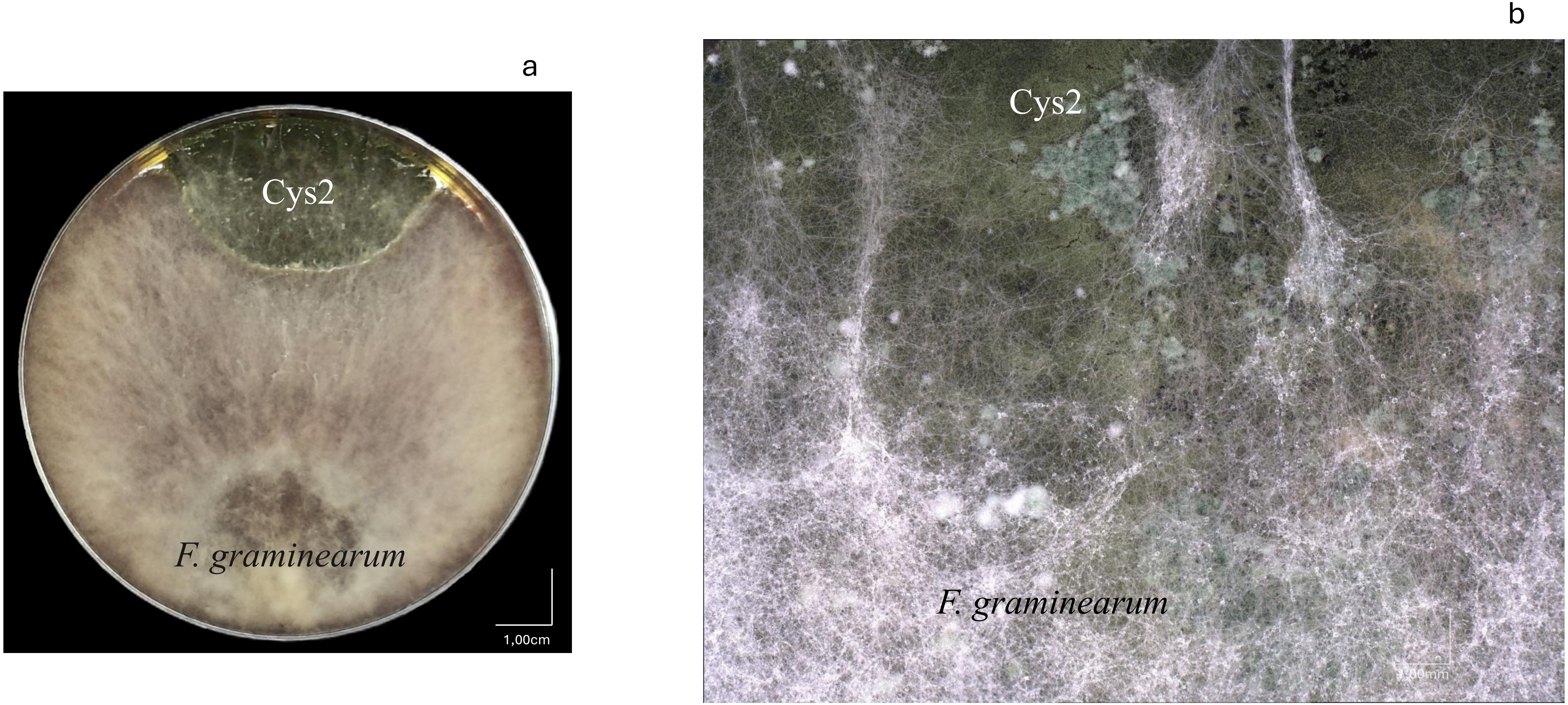

Confrontation (a) and magnification of the confrontation (b) between Cys2 (upper hyphae) and *S. sclerotiorum* (lower hyphae). *S. sclerotiorum* was not overgrown by Cys2. Images were taken after three weeks of growth at 25 °C ± 1.

a

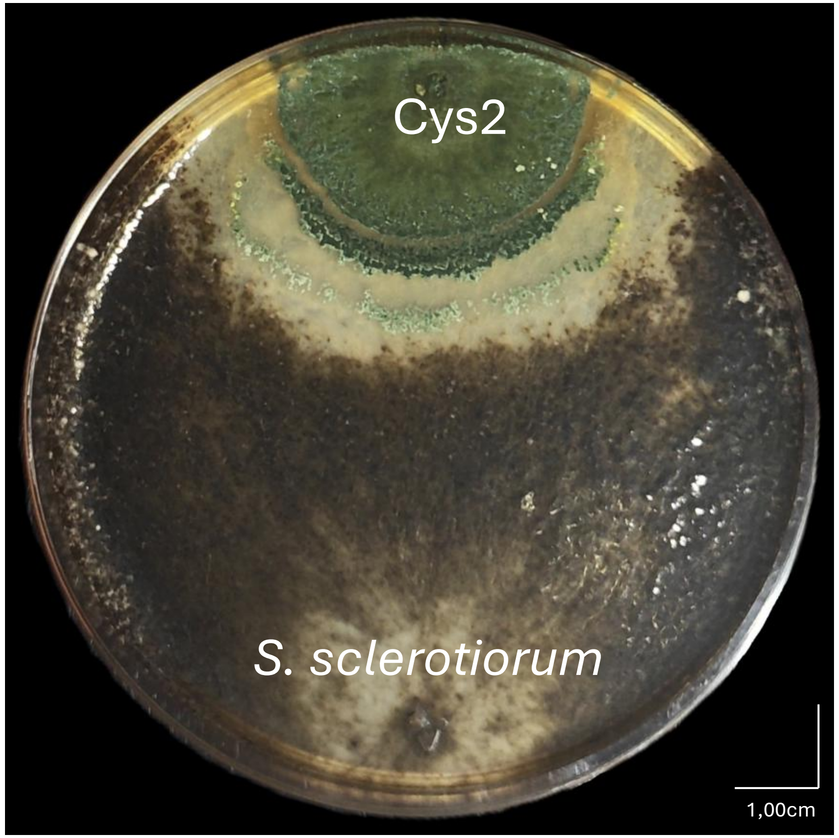

b

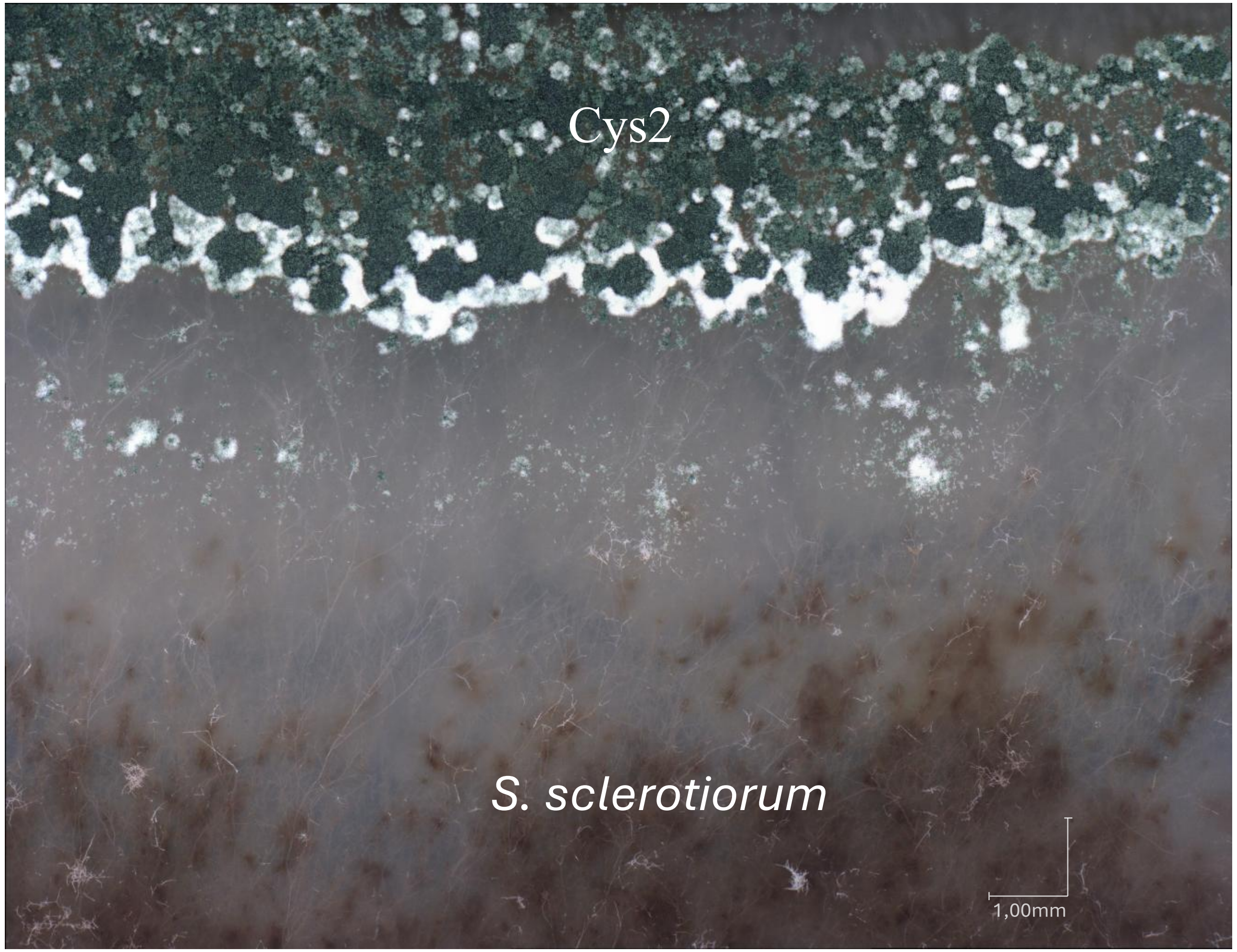

Supplement: Supplementary file 1 [file Data_Sheet_1.PDF]
